# Rapid Surveys Reveal Temporal Variation in Flowering Community Phenology in a Great Basin Desert Ecosystem

**DOI:** 10.1002/ece3.73340

**Published:** 2026-04-01

**Authors:** Megan E. Lahti, Eriko Sakamura, Minsung Jung, Cecilia M. Vigil, Kiley S. Smith, Megan Ramirez, Alec C. Brooks

**Affiliations:** ^1^ Biology & Public Health Department Truckee Meadows Community College Reno Nevada USA; ^2^ Computer Science and Technology Department Truckee Meadows Community College Reno Nevada USA; ^3^ Mathematics Department Truckee Meadows Community College Reno Nevada USA

**Keywords:** bimodal flowering season, climate change, sagebrush steppe, season, weather

## Abstract

Understanding temporal variation in flowering phenology is essential for predicting plant community responses to climatic change, especially in arid regions where moisture pulses drive productivity. We examined seasonal and interannual patterns of flowering phenology across 4 years (spring 2021–spring 2024) at the western margin of the Great Basin Desert along the Eastern Sierras ecotone (Reno, Nevada). Community flowering was strongly seasonal: spring supported nearly five times more flowering species than fall, consistent with deep winter–spring moisture recharge. Phenological timing varied across functional groups in spring, with herbaceous annuals and non‐native species showing more advanced flowering phenology than woody perennials and native species. In fall, communities showed only marginal phenological differentiation among lifespan groups. Interannual variation appeared to track climate conditions, with earlier flowering in cooler, wetter years and delayed flowering in warmer, drier years. Exploratory mixed‐effects models suggested that precipitation received the strongest relative support among candidate predictors, although climate variables overall provided limited explanatory power. Our results demonstrate that rapid surveys can detect phenological shifts and functional group responses, offering an efficient monitoring tool for arid landscapes with bimodal flowering seasons or limited monitoring capacity. Because the Reno‐Sparks region is among the fastest‐warming areas in the United States and lies at the cusp of the Eastern Sierras ecotone, these baseline data provide a critical foundation for tracking future community‐level shifts in flowering plant phenology.

## Graphical abstract

Across four years of surveys in an arid Great Basin ecosystem, community flowering phenology showed strong seasonal, interannual, and trait‐based variation, with most differences occurring in spring and little variation in fall. Flowering patterns broadly corresponded with interannual climate variability, consistent with the importance of moisture availability in arid ecosystems. Rapid, single‐visit community surveys provided an efficient framework for detecting phenological change in one of the fastest‐warming regions of the United States.

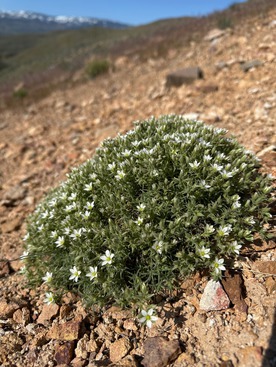

## 1 Introduction

Phenology is the seasonal timing of biological events and therein a fundamental ecological process tightly coupled to climatic cycles (Park and Post 2022). Because temporal cues are important to a species' life‐history, plant phenology serves as an indicator of climate change, wherein even modest shifts in timing can alter ecological interactions, resource availability, and ecosystem functioning (Menzel et al. 2006; Parmesean 2007; Sherry et al. 2007; Terasaki Hart et al. 2025; Richardson et al. 2010; Zhang et al. 2004; Luo et al. 2007).

Flowering phenology is especially influential because it determines reproductive opportunity, shapes fitness, and mediates interactions with the plant–animal interaction network (Park and Post 2022; Elzinga et al. 2007; Forrest 2015; Rafferty et al. 2020). Climatic shifts that advance, compress, or delay flowering windows can disrupt pollinator synchrony (Kudo and Cooper 2019), alter competitive and facilitative interactions among co‐flowering species (Forrest et al. 2010), and affect community stability (Sherry et al. 2007). Additionally, prolonged flowering seasons can elevate airborne pollen loads, with implications for allergy and asthma incidence, an emerging public‐health concern in rapidly warming urban‐wildland ecotones such as the Reno‐Sparks region (Wan et al. 2002; Climate Central 2023; Luo et al. 2007; Primack et al. 2009; Balmaki et al. 2024; Poole et al. 2019). Phenological responses vary widely among species due to differences in life‐history traits, phylogeny, and environmental sensitivity (e.g., Matthews and Mazer 2015; Rafferty et al. 2020).

Arid and semi‐arid ecosystems exhibit particularly dynamic phenological behavior. In these regions, precipitation serves as the primary cue for reproduction (Beatley 1974; Bowers and Dimmitt 1994; Crimmins et al. 2010; Wolkovich and Cleland 2014), producing pulse‐driven flowering, strong interannual variability in abundance and timing, and high species‐level plasticity (Liancourt et al. 2012; Iler et al. 2017; Wolkovich et al. 2013). Despite their broad global distribution, arid ecosystems remain underrepresented in phenological research, and the mechanisms driving divergent flowering responses in these environments are not well understood (Rafferty et al. 2020).

The Great Basin Desert provides a valuable setting for addressing these research gaps in arid systems, particularly along its western margin where it transitions abruptly into the eastern Sierra Nevada. Reno, Nevada, located at this ecotone, is the fastest‐warming city in the United States, with projected temperature increases by the 2080s of 5.7°C in summer and 4.1°C in winter alongside rising precipitation by 18% in summer and 26.3% in winter, based upon ensemble means from downscaled global climate models under moderate‐to‐high emissions scenarios (Fitzpatrick and Dunn 2019; Climate Central 2023). Regional climate shifts are expected to alter the length, predictability, and variability of growing seasons, with cascading effects on flowering phenology and community structure (e.g., Sherry et al. 2007; Park and Post 2022).

Although long‐term flowering datasets are increasingly available, many focus on individual species or first and last flowering dates, and require extensive survey effort (e.g., Amano et al. 2010; López López et al. 2020). These approaches can exaggerate phenological shifts and obscure community‐level patterns, and they are particularly challenging to apply in arid systems where flowering is episodic, spatially heterogeneous, and difficult to monitor continuously (Liancourt et al. 2012; CaraDonna et al. 2014; Rafferty et al. 2020). Rapid, community‐level surveys conducted during peak flowering offer a complementary approach by capturing variation in flowering timing, diversity, and functional group composition across seasons and years (Denny et al. 2014).

Here, we use multi‐year rapid surveys to evaluate how peak flowering community phenology varies across seasons, years, and functional groups in a Great Basin Desert plant community situated near the Eastern Sierras ecotone. Specifically, we evaluate whether (1) community phenology varies among years and between seasons, (2) phenological timing differs among growth forms, lifespan categories, and species status, and (3) interannual climatic variation corresponds with patterns in community phenology. Because water availability is a primary limiting resource in arid ecosystems, we predict that flowering phenology varies across functional groups and most strongly tracks seasonal moisture availability, including precipitation and winter snow water content. By examining phenological dynamics in one of North America's fastest‐warming regions, this study demonstrates the utility of rapid survey methods for assessing how climate change may reshape flowering community structure in arid ecosystems.

## 2 Methods

### 2.1 Study Site

**FIGURE 1:**
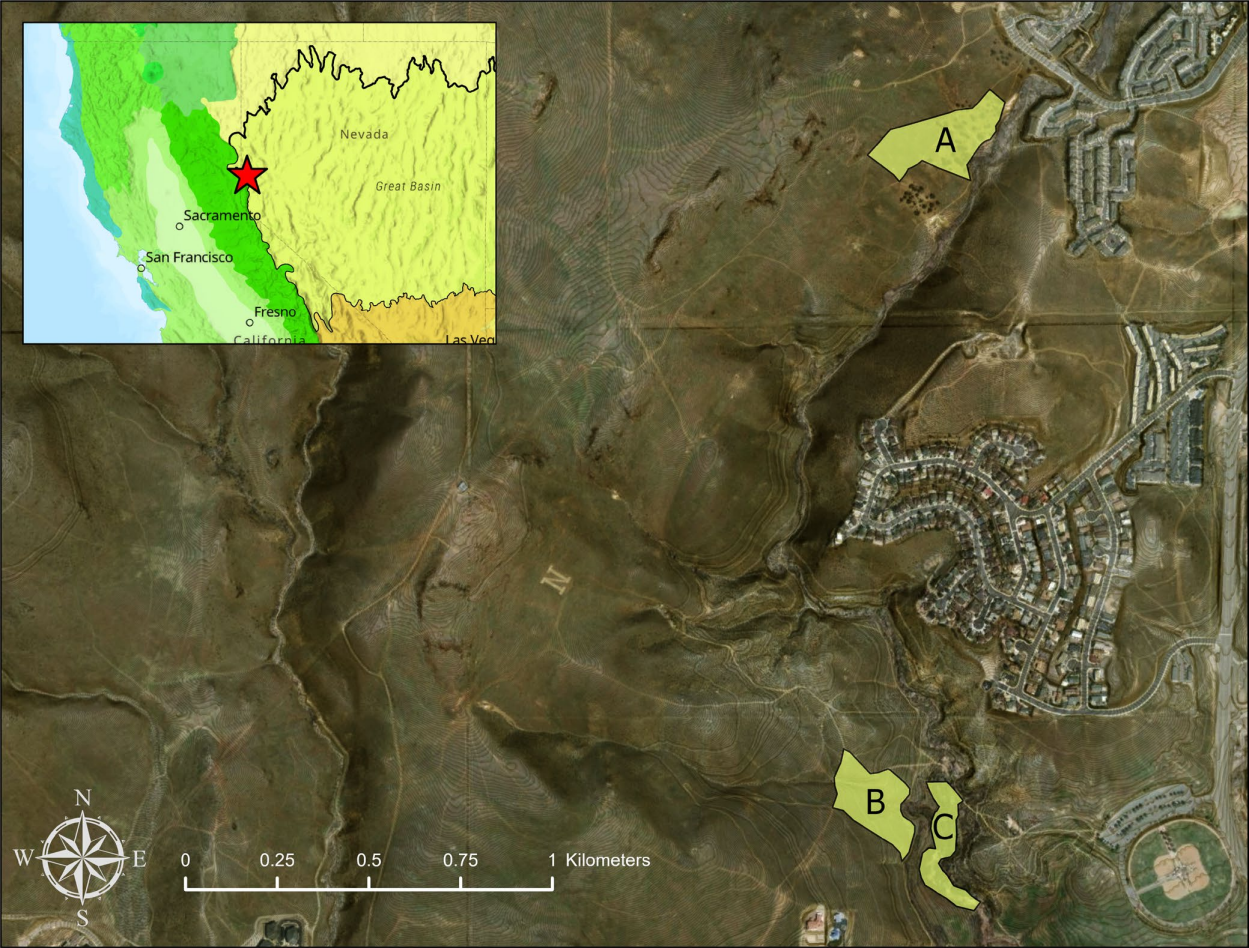
Study site and surveyed habitats at the western Great Basin Desert–Eastern Sierra ecotone (Reno, Nevada). We conducted single‐visit, peak flowering surveys in spring and fall from 2021 to 2024 across three habitat types: (A) forest/grassland, (B) sagebrush, and (C) riparian/wetland. Because flowering phenology did not differ significantly among habitats within seasons, data from all survey plots were pooled to represent maximum seasonal flowering diversity.

The study was conducted in urban open space habitat in the Peavine Peak foothills of Reno, Nevada (Figure 1). The open space area is bisected by Evans Canyon, which contains an ephemeral stream (Evans Creek) supporting a mosaic of permanent riparian, semi‐permanent wetland, and upland sagebrush steppe, grassland, and forest habitats. Historically, the area was grazed by cattle and sheep until around 1994, while fire disturbance has been absent for at least 25 years (Washoe County 2025; National Interagency Fire Center 2025). At present, human activity in this open space area is largely limited to hiking and biking along trails, with residential development bordering the northern and eastern edges.

The study site occurs at the western edge of the Great Basin Desert along the ecotone with the Eastern Sierra ecosystem. The cold semi‐arid climate for this region is classified as BSk, under the Köppen‐Geiger system (Peel et al. 2007), typical of the Great Basin Desert. Summers are hot and dry with mean monthly maximum temperature near 33°C and < 5 mm/month precipitation, whereas winters are cold, with mean monthly minimum temperatures around −3°C. Precipitation is seasonal and predominantly occurs as snowfall during winter (January peak ~74 mm/month) and rainfall during spring (~20 mm/month; National Oceanic and Atmospheric Administration [NOAA] 2024).

### 2.2 Vegetation Communities

Flowering phenology surveys were conducted in three survey plots within the open space area, encompassing three vegetation communities: (1) forest/grassland, corresponding to Great Basin & Inter‐mountain Introduced Annual Grassland, (2) sagebrush, corresponding to Inter‐Mountain Basins Big Sagebrush Shrubland, and (3) riparian/wetland, corresponding to Great Basin Foothill and Lower Montane Riparian Woodland (Peterson 2008; LandFire 2025). Physical site characteristics including area, slope, aspect, elevation, and soil composition are provided in Table 1.

**TABLE 1:** Survey plot physical characteristics.

Habitat	Study plot area (ha)	Elevation (m)	Slope (%)	Aspect	Parent material	Surface substrate	Surface substrate depth (cm)
Forest/Grassland	438	1495	15–30	SE	Colluvium/residuum	Gravelly, sandy loam	0–20
Sagebrush	350	1454	23	E	Colluvium/residuum	Cobbly, sandy loam	0–8
Riparian/Wetland	238	1432	32	SE	Mixed alluvium	Gravelly loam	0–31

*Note:* All study plots occur within 1.4 km of each other and have similar profiles. Site profiles are obtained from the United States Department of Agriculture Natural Resources Conservation Service Web Soil Survey.

### 2.3 Flowering Phenology Surveys

We conducted seven phenology surveys, once per season (spring and fall) each year from spring 2021 to spring 2024. The timing of these surveys corresponded to the bimodal seasonal moisture regime characteristic of the region (NOAA 2024; Snyder et al. 2016; Ogle and Reynolds 2004). Each survey was scheduled to coincide with the anticipated peak flowering period within each season, determined from prior site observations and regional flowering records. One survey was performed per habitat and season, and thus each dataset represents a phenological snapshot of community flowering rather than a continuous flowering curve. To ensure sampling consistency for interannual comparison, surveys followed a fixed weekday schedule and were conducted on the same Friday each year, irrespective of calendar date. Spring surveys occurred on 07 May 2021, 06 May 2022, 05 May 2023, and 04 May 2024, while fall surveys occurred on 01 October 2021, 30 September 2022, and 29 September 2023 between 09:00 and 16:00 h and at temperatures > 15°C.

**FIGURE 2:**
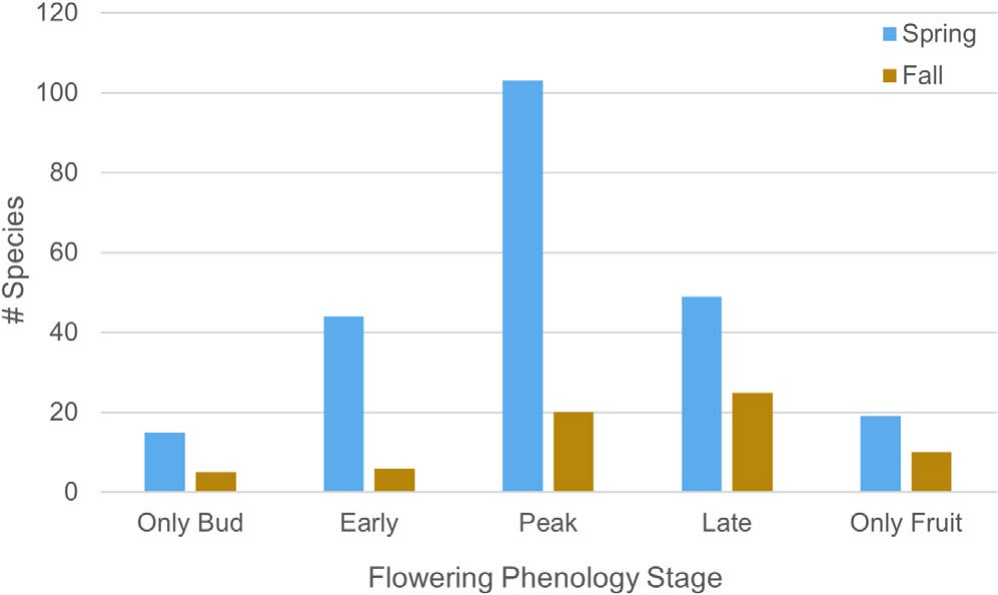
Seasonal distribution of flowering phenology. Across seasons, surveys coincided with peak flowering. Spring surveys additionally coincided with absolute peak flowering activity. Although normality tests for fall absolute peak flowering activity did not detect significant deviations from normality, visual inspection of the Q–Q plot indicated that the fall surveys were conducted post‐absolute‐peak. We report statistical results for fall, but suggest caution when interpreting spring‐fall comparisons of absolute peak flowering. Flowering phenology values represent sequential reproductive stages: 1 = buds only, 2 = early flowering, 3 = peak flowering, 4 = late flowering, 5 = fruiting (flowers absent).

Flowering phenology was classified into five categorical stages: (1) flower buds only (flower buds present but not open), (2) early flowering (open flowers present but not dominant across reproductive structures), (3) peak flowering (open flowers dominant across reproductive structures), (4) late flowering (senescent flowers dominant across reproductive structures), and (5) fruits only (flowers absent; immature or mature fruits present). Because our surveys intentionally capture maximum flowering community diversity and flowering time across species is variable, we define peak flowering as the period when the majority of species within the flowering community are in open flower, including early, peak, and late flowering (Stages 2–4). We define absolute peak flowering as species whose flowering phenology is limited to peak flowering (Stage 3). Spring surveys coincided with the community peak and absolute flowering, while fall surveys only coincided with the community peak flowering period (Figure 2). Normality tests for fall phenological distributions did not detect deviations from normality for peak flowering (Shapiro–Wilk: W = 0.88, *p* = 0.32; KS test: *D* = 0.24, *p* = 0.87), but the small sample size (*N* = 5 categories) limits statistical power. Visual inspection of the histogram and Q–Q plot indicated non‐normal patterns consistent with surveys occurring just after absolute peak flowering. Thus, we suggest caution when interpreting spring‐fall statistical comparisons of absolute peak flowering due to biases that may result from seasonal differences in the absolute peak flowering window timing.

During each survey, trained volunteers systematically searched the designated study plants for at least 30 min, recording all flowering species and assigning each to the appropriate phenological category. For each species, phenological stage was assigned based on observations from ≥ 30 individuals, and the modal flowering stage was recorded as the species‐level phenology value. Because flowering stages represent a phenological continuum, cases where two adjacent phenology stages were equally frequent were interpreted as transitional. In these cases, the phenology score was recorded as the mean of the two stages (0.5 increments). True bimodal flowering (non‐adjacent phenological peaks) was not observed in the data set. Fruits were only recorded if they resulted from flowering activity within that same season. For morphologically similar taxa that could not be confidently distinguished in the field, observations were combined at the genus level, and their phenological data averaged. Following each survey, all records were verified and cross‐checked by the survey lead (MEL) to minimize non‐sampling error and validate the dataset.

### 2.4 Analyses

All statistical analyses were conducted in R (R Core Team 2024), and maps were produced using ArcGIS Pro software (ESRI 2025). Our primary goal was to evaluate seasonal and interannual variation in community‐level flowering phenology and composition. Because our emphasis was on whole‐community responses rather than microhabitat‐specific patterns, and preliminary tests showed no meaningful phenological differences among habitats (*p* > 0.05), data from the three survey plots were pooled to maximize species diversity within each season. To ensure consistency in sampling effort and species composition, we limited analyses to flowering species represented by ≥ 30 individuals per survey and present in all interannual spring or fall surveys. A total of 23 species were included in spring analyses, and a total of seven species were included in fall analyses. To verify that the pooled dataset adequately captured species richness, we generated within‐season species effort curves (SEC) using a presence/absence matrix with 500–1000 random permutations and 95% confidence intervals. Curve asymptotes were used to assess sampling completeness for both seasons.

Patterns of species turnover among years, seasons, and transects were quantified using the Jaccard similarity index. For each transect × season × year combination, presence/absence was summarized, and Jaccard dissimilarity was computed with vegdist (method = “jaccard,” binary = TRUE) in the vegan package (Oksanen et al. 2025). Similarity was expressed as 1 − dissimilarity and used to compare within‐ vs. between‐season, within‐ vs. between‐year, and within‐ vs. between‐transect community similarity.

Interannual differences in flowering phenology within each season were tested using Friedman tests, with habitats treated as blocks and years as repeated measures. This nonparametric approach was chosen because it does not assume normality and is appropriate for non‐normally distributed data where multiple measurements are made within the same groups. When Friedman tests were significant (*α* = 0.05) or indicated weak or emerging trends (0.05 < *p* ≤ 0.10), pairwise Nemenyi post hoc comparisons were conducted using PMCMRplus (Pohlert 2024).

To evaluate multivariate shifts in phenological structure, we conducted a PERMANOVA (vegan package), using 999 permutations and Euclidean distances of phenology values (Oksanen et al. 2025). For each season, we fit two‐factor models with year and transect as predictors, restricting permutations within transects using the strata argument to account for repeated measurements. Pairwise PERMANOVA comparisons among years were performed, with *p*‐values adjusted for multiple comparisons.

To assess variation in phenology among functional groups, we analyzed differences across growth form (forb, grass, shrub, tree) and lifespan categories (annual herbaceous, herbaceous biennial, perennial herbaceous, perennial mixed, perennial woody) following the Biota of North America Program (Kartesz 2015). We also included species status (native vs. non‐native) following Wolkovich and Cleland (2014) which defines non‐native any species whose presence is outside of its established home range, regardless of its impact on the native ecosystem. Because phenology values were non‐normally distributed, we used Kruskal–Wallis tests for multi‐group comparisons (growth form and lifespan) followed by Dunn's post hoc tests with Holm correction, and Wilcoxon rank‐sum tests for two‐group comparisons (species status).

Climate analyses were conducted in two stages. First, we evaluated interannual differences in seasonal weather conditions preceding each survey to provide environmental context for observed variation in flowering phenology. Seasonal climate variables (minimum and mean temperature, precipitation, snow water content) from the 8 weeks preceding each survey were analyzed to characterize interannual climatic variation among survey years (NOAA 2024; California Department of Water Resources 2024). We evaluated ANOVA assumptions using Shapiro–Wilk tests for normality and Levene's tests for homogeneity of variance. When assumptions were met, we used one‐way ANOVA with Tukey's HSD; when violated, we used Kruskal–Wallis tests followed by pairwise Wilcoxon tests with Holm correction.

Second, to evaluate potential climate–phenology relationships, we focused on spring surveys, when climatic controls on germination and early development are expected to exert the strongest influence on flowering timing (Pareja‐Bonilla et al. 2025; Jiang et al. 2025). Climatic predictors were calculated to represent cumulative thermal and moisture conditions influencing plant development, including chilling (degree‐days < 0°C), forcing accumulation (degree‐days > 0°C), freezing exposure (number of days with minimum temperature < 0°C), and precipitation totals. Climatic metrics were calculated across an accumulation period spanning 01 October of the preceding year through 4 weeks prior to each survey date. This extended accumulation window was used to capture climatic conditions influencing early developmental processes across multiple species, whose germination, dormancy release, and flowering initiation occur at different times within the community, making species‐specific chilling and forcing windows difficult to define (Körner and Basler 2010; Jiang et al. 2025). Alternative thermal threshold values were evaluated prior to model fitting. Forcing degree‐days calculated above 0°C and above 5°C were strongly correlated across the 4 study years (Pearson *r* = 0.95), as were chilling degree‐days calculated below 0°C and below 5°C (*r* = 0.95). Because these alternative metrics captured nearly identical interannual temperature patterns, only the 0°C threshold formulations were retained to avoid redundant predictors within the candidate model set.

Analyses were restricted to the 23 plant species observed in all survey years to allow within‐species comparisons across years. Associations between climate variables and spring flowering phenology were evaluated using linear mixed‐effects models (LMMs), with species included as a random intercept to account for repeated observations of species across survey years, while climatic predictors varied at the year level. Individual climatic variables were evaluated as fixed effects using separate single‐predictor mixed‐effects models, and candidate models were compared using Akaike's Information Criterion (AIC). Because spring observations consisted of only four annual surveys, analyses were treated as exploratory assessments of emerging climate–phenology patterns rather than confirmatory hypothesis tests. Model support was interpreted using ΔAIC values, with models within ΔAIC ≤ 2 considered similarly supported. Given the limited temporal replication, inference emphasized effect direction, relative model support, and consistency among predictors rather than formal statistical significance testing. All LMM analyses were conducted using the lme4 package (Bates et al. 2015).

## 3 Results

### 3.1 Phenology Structure

**FIGURE 3:**
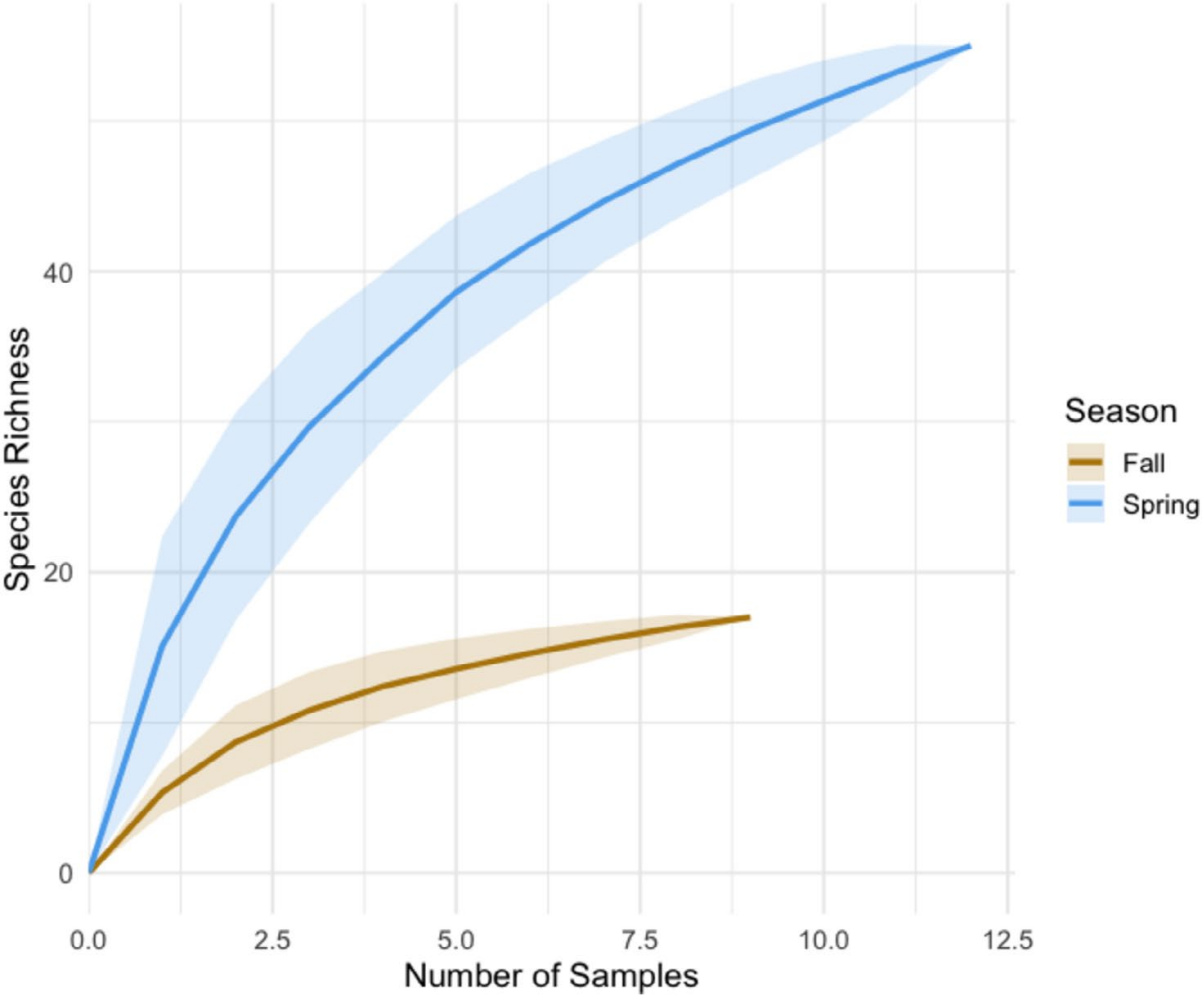
Species–effort curves (SECs) with 95% confidence intervals for flowering species richness across seasons. Because flowering phenology did not differ significantly among habitats, surveys from all survey plots (forest/grassland, sagebrush, riparian/wetland) were pooled within each season. The SECs therefore represent maximum seasonal flowering diversity across the study area. Both curves approach an asymptote, with substantially higher species richness in spring than in fall.

**FIGURE 4:**
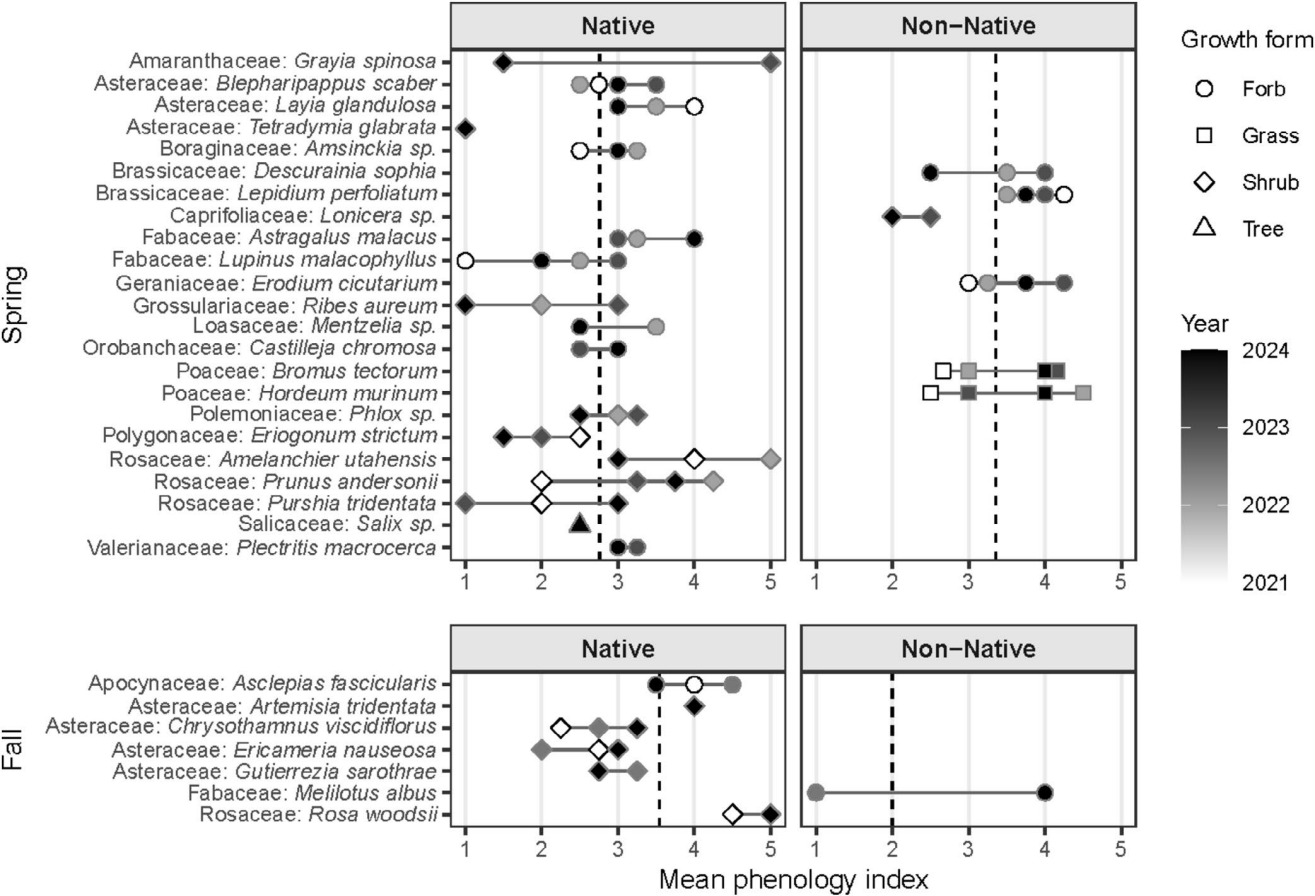
Species‐level flowering diversity across seasons. Among species observed during all within‐season surveys, both phenological variation and species richness were higher in spring than in fall. Within spring, native species showed lower phenological indices and reduced phenological variation compared to non‐native species. Flowering phenology values represent sequential reproductive stages: 1 = buds only, 2 = early flowering, 3 = peak flowering, 4 = late flowering, 5 = fruiting (flowers absent).

The SEC for both seasons indicates that the flowering community richness was adequately sampled to include most species (Figure 3). Species richness plateaus near 60 species in spring and near 20 species in fall, indicating seasonal variation in species diversity. Across habitats, 27 plant families and 73 species were observed (Table S1). Spring surveys included 25 families and 56 species and fall surveys included 8 families and 18 species. Collectively, most of these species were forbs (57.8%) or shrubs (33.3%). Of the 19 invasive species observed, most (68.4%) occur in spring compared to fall (31.6%). Species exhibited broad variation in mean flowering phenology, particularly in spring when community diversity was greater (Figure 4).

Across survey plots, flowering species had a high level of spatial heterogeneity (*J* = 0.17–0.20), with riparian/wetland habitat having the highest diversity, and thus greatest distinction from forest‐grassland and sagebrush habitats. Within‐season variation showed moderate temporal stability among flowering species (*J* = 0.608), and there was complete seasonal turnover between spring and fall (*J* = 0; *F* = 7.91, df = 1, *p* < 0.001, *R* ^2^ = 0.30).

### 3.2 Phenological Patterns Across Functional Groups

**FIGURE 5:**
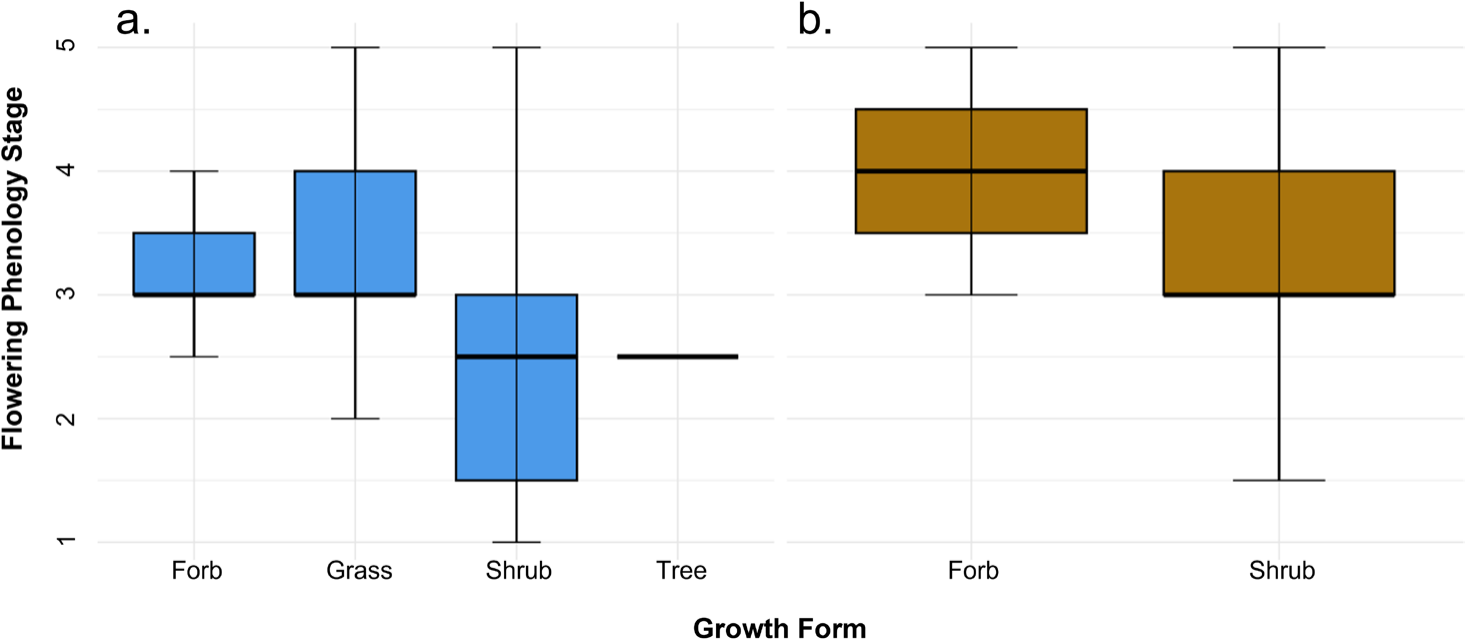
Seasonal distributions of flowering phenology among growth forms. Flowering phenology differed significantly among growth forms in (a) spring but not in (b) fall. In spring, forbs and marginally grasses showed more advanced flowering phenology than shrubs. Grasses showed a similar trend toward more advanced phenology than trees, although this difference was only marginally significant. Flowering phenology values represent sequential reproductive stages: 1 = buds only, 2 = early flowering, 3 = peak flowering, 4 = late flowering, 5 = fruiting (flowers absent).

Growth form significantly influenced flowering phenology (χ^2^_(3)_ = 12.64, *p* = 0.005), with variation driven primarily by spring patterns (Figure 5). In spring, shrubs exhibited younger phenological stages (mean = 2.54, median = 2.50) than forbs (mean = 3.15, median = 3.00; *Z* = 3.23, *p* = 0.004) and grasses (mean = 3.47, median = 3.00; *Z* = 3.10, *p* = 0.005). Trees showed a similar trend (mean = 2.50, median = 2.50) with grasses, though differences were weaker (*Z* = 2.25, *p* = 0.049). No significant differences among growth forms were detected in fall.

**FIGURE 6:**
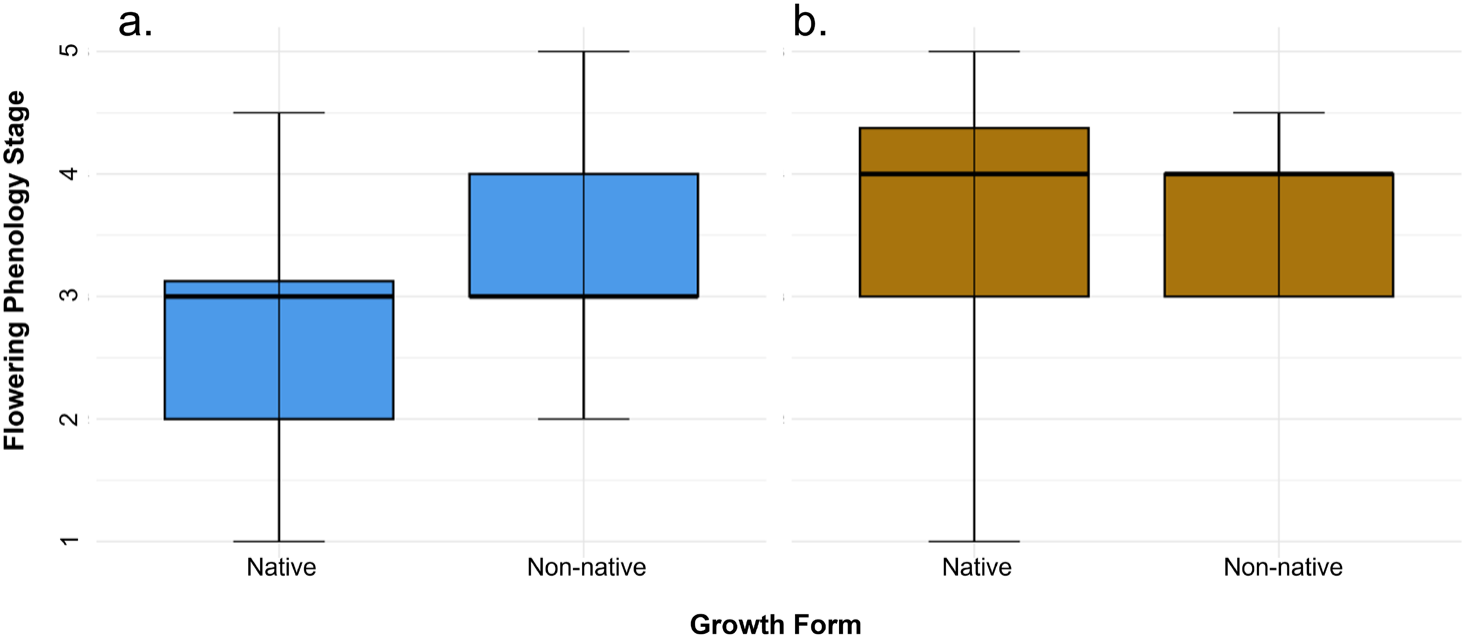
Seasonal distributions of flowering phenology for native and non‐native species. In (a) spring, non‐native species had a significantly advanced flowering phenology than native species. In (b) fall, phenology stages did not differ significantly between native and non‐native species. Flowering phenology values represent sequential reproductive stages: 1 = buds only, 2 = early flowering, 3 = peak flowering, 4 = late flowering, 5 = fruiting (flowers absent).

Species status also affected flowering phenology (*U* = 3556, *p* = 0.002), with non‐native species exhibiting advanced phenological stages compared to native species (Figure 6). This distinction occurred only during the spring (*W* = 924, *p* < 0.001), when non‐native taxa reached more advanced floral stages (mean = 3.47, SD = 0.78, median = 3.50) than native taxa (mean = 2.82, SD = 0.98, median = 3.00).

**FIGURE 7:**
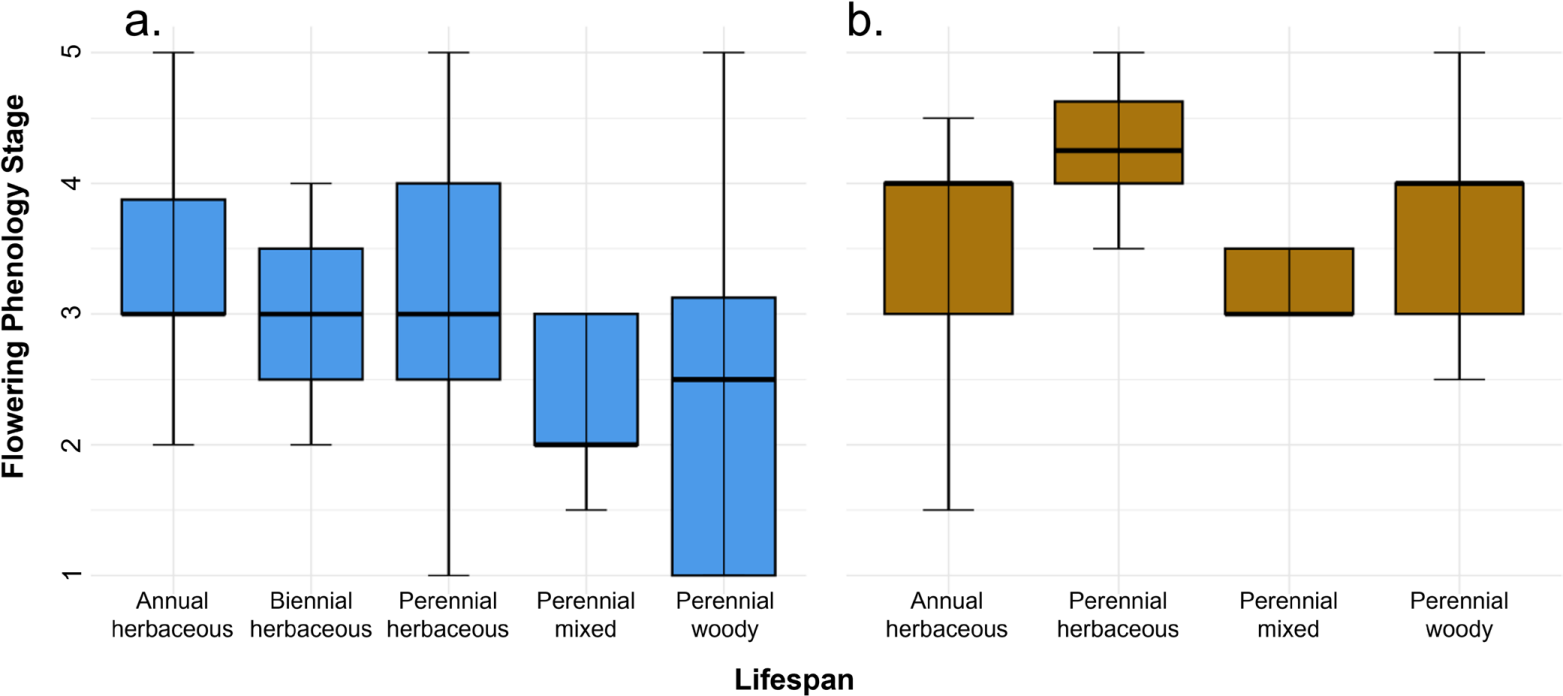
Seasonal distributions of flowering phenology across plant lifespan types. In (a) spring, annual herbaceous species had significantly advanced flowering phenology than perennial woody species and marginally advanced than perennial mixed species. Perennial herbaceous species also had marginally advanced phenology values than perennial woody species. In (b) fall, phenological differences among lifespan groups were weak and only marginally significant. Flowering phenology values represent sequential reproductive stages: 1 = buds only, 2 = early flowering, 3 = peak flowering, 4 = late flowering, 5 = fruiting (flowers absent).

Lifespan exhibited the same seasonal pattern, with stronger differentiation in spring than in fall (Figure 7). Across seasons, flowering phenology varied significantly with lifespan (χ^2^_(4)_ = 11.23, *p* = 0.02), driven by annual herbaceous species having more advanced flowering phenology than perennial mixed (*p* = 0.049) and marginally more advanced than perennial woody (*p* = 0.05). In spring, the effect was more pronounced (χ^2^_(4)_ = 15.943, *p* = 0.003), as annual herbaceous species had more advanced flowering phenology than perennial woody species (*p* = 0.004) and marginally more advanced than perennial mixed (*p* = 0.06). Similarly, perennial herbaceous flowering phenology was marginally more advanced than perennial woody growth forms (*p* = 0.087). In fall, phenological differences among lifespan groups were weak and only marginally significant (χ^2^_(3)_ = 7.05, *p* = 0.07).

### 3.3 Temporal Variation in Phenology and Climate Conditions

Interannual variation in flowering phenology was significant across seasons (*F*_(1,224)_ = 8.99, *p* = 0.002, *R* ^2^ = 0.04; Table 2). In spring, phenological stages varied marginally among years (χ^2^_(3)_ = 7.00, *p* = 0.07), with the greatest difference between 2021 and 2023 (*q* = 5.87, *p* = 0.055). Spring 2021 exhibited the lowest phenology values (mean = 2.81, SD = 0.98, median = 3.00), corresponding with the driest winter and the warmest, driest spring during the study period. In contrast, spring 2023 exhibited the highest phenology values (mean = 3.10, SD = 0.95, median = 3.00), occurring during the wettest winter and a comparatively cool, wet spring. In fall, a similar marginal trend was detected (χ^2^_(2)_ = 4.67, *p* = 0.097), with the strongest difference occurring between 2022 and 2023 (*q* = 2.89, *p* = 0.10). Fall 2022 had the lowest phenology values (mean = 3.21, SD = 1.34, median = 3.25), corresponding with the warmest fall during the study period. In contrast, fall 2023 exhibited the highest phenology values (mean = 3.80, SD = 0.73, median = 4.00), following the coolest and wettest fall conditions.

**TABLE 2:** Flowering phenology across years and seasons in relation to interannual climate variability.

		Spring				Fall		
		2021	2022	2023	2024	2021	2022	2023
Phenology	# Species	23				7		
	Mean	2.81†	3.16	3.10^†^	2.89	3.25	3.21^†^	3.8^†^
	SD	(0.98)	(0.99)	(0.95)	(0.96)	(1.05)	(1.34)	(0.73)
Snow WC (mm)	Mean	221.92ᵃ	355.85ᵇ	1065.37ᶜ	472.61ᵃ	—	—	—
	SD	(137)	(92)	(82.5)	(72.0)	—	—	—
Precipitation (mm)	Mean	0.02ᵃ^†^	0.11ᵃ	1.18ᵇ^†^	0.55ᵃ	0.11	0.17	0.43
	SD	(0.12)	(0.63)	(3.15)	(1.88)	(0.47)	(0.64)	(1.37)
Mean Temperature (°C)	Mean	2.51ᵃ	2.21ᵃᵇ	−0.05ᵇ	1.44ᵇ	12.70ᵃ	13.67ᵃ	10.56ᵇ
	SD	(4.74)	(3.91)	(4.88)	(13.79)	(5.12)	(4.39)	(14.02)
Minimum Temperature (°C)	Mean	−2.27	−2.05	−4.46	1.42	7.57	8.88	6.29
	SD	(4.39)	(4.12)	(5.06)	(4.00)	(4.79)	(4.15)	(3.04)

*Note:* Within‐season interannual variation in phenology was marginally significant in both seasons. In spring, phenology index values were lowest following the driest winter and warmest, driest season (2021) and highest following the wettest winter and the coolest, wettest season (2023). In fall, the greatest difference in phenology occurred between 2022 and 2023 and reflected a similar pattern where phenology values were lowest following the warmest season (2022) and highest following the coolest and wettest season (2023). Seasonal climate variables are presented as descriptive indicators of interannual conditions and are not intended to represent mechanistic chilling or forcing metrics. Superscript letters represent post hoc grouping results (Dunn test) for each weather variable within each season; years sharing a letter do not differ significantly. Superscript symbols indicate significance levels, with all comparisons significant at *p* < 0.05 except for ^†^(*p* ≤ 0.10).

There were no significant differences in flowering community phenological structure among survey plots, years, or their interaction during spring (*p* > 0.05). However, in fall, year had a marginally significant effect on phenological composition (*F*_(1,47)_ = 4.32, *p* = 0.047, *R* ^2^ = 0.08). Although post hoc pairwise comparisons showed no significant differences between years, unadjusted *p*‐values suggested an emerging shift in 2023, with marginal dissimilarities between 2021 (*p* = 0.087) and 2022 (*p* = 0.112).

Seasonal climatic conditions preceding spring surveys were broadly similar among years, with moderate interannual variation in precipitation and chilling accumulation (Table 3). Alternative mixed‐effects model structures that included species as a random intercept, with or without an additional year random effect, produced similar parameter estimates and model rankings. Across candidate models evaluating climate–phenology relationships, all models received comparable support (ΔAIC ≤ 2), including the null model lacking climatic predictors (Table 4), indicating limited statistical separation among predictors. Among models including climatic predictors, the precipitation model produced the lowest AIC value, although support was nearly indistinguishable from that of the null model (ΔAIC = 0.32). Under the species + year random‐effects structure, the null model had the lowest AIC, followed by the precipitation model ranking next (ΔAIC = 1.01); however, all candidate models fell within ΔAIC ≤ 2.

**TABLE 3:** Seasonal climatic conditions preceding spring phenology surveys.

Year	Precipitation (mm)	Cold‐season conditions		Thermal forcing
	Full season	Chilling (DD < 0°C)	Freezing (days)	(DD > 0°C)
2021	60.0	−13.6	131	1135.8
2022	171.0	−36.1	110	1227.3
2023	284.6	−106.5	139	863.1
2024	141.6	−20.2	97	1252.8

*Note:* Climatic predictors represent accumulated thermal and moisture conditions calculated from 01 October of the preceding year through 4 weeks prior to each survey date. Metrics include chilling accumulation degree‐days (degree‐days < 0°C), freezing exposure (number of days with minimum temperature < 0°C), forcing accumulation (degree‐days > 0°C), and total precipitation. Climatic variables were derived from daily weather observations from the local NOAA weather station.

**TABLE 4:** Candidate linear mixed‐effects models evaluating associations between climatic predictors and spring community flowering phenology.

	AIC	ΔAIC	*wᵢ*	*β*	SE	*t*	df	*p*
**Random‐effects: species only predictor**								
Null	345.49	0.00	0.28	—	—	—	3	—
Precipitation	345.81	0.32	0.24	0.0010	0.0008	90.13	4	0.196
Chilling	346.51	1.02	0.17	0.0016	0.0017	92.01	4	0.324
Freezing	347.41	1.92	0.11	0.0010	0.0035	93.98	4	0.776
Forcing	347.47	1.98	0.10	−0.0001	0.0004	93.34	4	0.895
**Random‐effects: species + year predictor**								
Null	346.23	0.00	0.31	—	—	—	4	—
Precipitation	347.24	1.01	0.19	0.0010	0.0010	4.13	5	0.349
Chilling	347.66	1.43	0.15	0.0017	0.0022	3.81	5	0.483
Freezing	348.20	1.97	0.12	0.0009	0.0050	3.69	5	0.866
Forcing	348.22	1.99	0.12	−0.0001	0.0006	3.59	5	0.913

*Note:* Two random‐effects structures were evaluated: Species only and species + year. Models are ranked by Akaike's Information Criterion (AIC), with ΔAIC representing the difference from the best‐supported model within each random‐effects structure. Akaike weights (*wᵢ*) indicate the relative support for each model within the candidate model set. Models with ΔAIC ≤ 2 are considered to have comparable support. Model interpretation is exploratory due to the limited temporal replication of survey years.

## 4 Discussion

This study examined temporal, functional, and climatic variation in community flowering phenology in an arid ecosystem of the Great Basin Desert. Community phenology varied among years and between seasons, consistent with the region's bimodal moisture regime. Phenological timing also differed among functional groups, suggesting that flowering schedules reflect life‐history strategies associated with growth form, lifespan, and species status. In spring, interannual differences in community phenology broadly paralleled observed variation in pre‐season climate conditions, with more advanced flowering stages generally occurring in cooler, wetter years and lower stages during warmer, drier conditions. Exploratory mixed‐effects models evaluating potential climate–phenology relationships showed limited support for climatic predictors, with all candidate models receiving similar support to the null model. Despite temporal limitations, rapid, peak‐season community surveys effectively captured broad temporal and functional patterns in flowering dynamics. Together, these findings highlight that climatic variability and plant functional traits may interact to shape seasonal community‐level flowering dynamics in a rapidly warming arid ecosystem.

### 4.1 Phenology Structure

Although species richness varied across microhabitats, flowering stages did not, suggesting that phenological timing in this system is driven by regional climatic cues rather than local microhabitat conditions. The riparian–wetland plot supported the highest diversity, consistent with the expectation that moisture‐rich areas sustain greater biodiversity in arid landscapes (Bennett et al. 2014; Shaw and Cooper 2008). Diverse plant assemblages are often characterized by staggered flowering schedules that buffer community‐level phenology through temporal niche partitioning, as shown in other ecosystems (Chesson 2000; Dorado and Vázquez 2016; Wolf et al. 2017; Rathcke and Lacey 1985; Iler et al. 2017). Such stabilizing dynamics may operate at finer temporal resolutions or outside the peak flowering window captured by our rapid survey method, and therefore may not be detectable with our rapid survey approach.

Community composition differed sharply between seasons, consistent with the bimodal bloom season characteristic of the Great Basin Desert, which reflects the region's precipitation pulses in winter–spring and late‐summer. Both biotic (pollination, competition, herbivory) and abiotic (drought, freezing) pathways influence, and are influenced by, phenology and community structure (Wolkovich and Cleland 2014; Forrest et al. 2010; Wolf et al. 2017). For example, reduced diversity can increase soil temperature and decrease moisture, advancing flowering in ways comparable to regional warming (Wolf et al. 2017). Although our surveys were not designed to isolate these mechanisms, the strong seasonal structuring in our dataset suggests that community‐level phenology in this system emerges from interacting biotic and abiotic forces whose relative importance likely shifts between seasons.

Similarly, clade‐specific evolutionary histories also shape phenological cueing (Davies et al. 2013). In unpredictable environments, some lineages, such as desert annuals in the Asteraceae or Brassicaceae families, track precipitation pulses or photoperiod more strongly than temperature, particularly in late‐season when dominant environmental cues shift (Beatley 1974; Davis et al. 2010; Wolkovich and Cleland 2014). Evidence from other seasonally complex systems supports this pattern: in a tropical deciduous forest with bimodal precipitation, phylogenetic effects corresponded with biotic traits related to flowering duration, whereas variation in flowering timing, the focus of our study, was primarily driven by abiotic cues such as precipitation regime and photoperiod (Cortés‐Flores et al. 2017). In our dataset, Asteraceae species showed less interannual variation in flowering stage than Rosaceae, suggesting more consistent phenological timing in spring, though these trends remain tentative given limited taxa and years. Together, lineage‐specific patterns mediate the interplay between evolutionary history and local environmental conditions in shaping community phenology (Song et al. 2025; Wolkovich and Cleland 2014).

### 4.2 Functional Group Differences

Growth form and lifespan are inherently correlated, but not interchangeable. Growth form reflects a plant's architectural strategy, whereas lifespan captures additional temporal strategies that are often more complex and multidimensional (Salguero‐Gómez et al. 2016). Reflecting this complexity, plant ecology has increasingly shifted from a species‐centric to a trait‐centric framework (Lavorel and Garnier 2002; Klimešová et al. 2016). In our study, similar spring patterns across growth form and lifespan, including more advanced flowering phenology in annual forbs and grasses than in perennial shrubs and trees, are consistent with well‐known contrasts in rooting depth and water use. Herbaceous species with shallow roots track rainfall closely and respond rapidly to surface moisture, a pattern also documented in tropical systems where herb phenology aligns tightly with the rainy season (Cortés‐Flores et al. 2017). In contrast, woody species can decouple flowering from short‐term moisture availability by relying on deep roots and stored water, allowing some to flower even during dry periods (Cortés‐Flores et al. 2017). In arid systems, winter precipitation recharge further enhances spring bloom, particularly for deeper‐rooted perennials (Comstock and Ehleringer 1992; Crimmins et al. 2010). By fall, phenological differences across both functional traits were diminished or absent, likely in response to late‐season declines in soil moisture and temperature shifts which compress the bloom window for all species.

Species status also contributed to phenological differentiation. Non‐native species reached more advanced flowering stages in spring than native species, consistent with the greater phenological flexibility and climatic responsiveness often observed in non‐native taxa (Wolkovich et al. 2013; Wolkovich and Cleland 2014; Willis et al. 2010; Anderson et al. 2012). In the Great Basin, this pattern mirrors the accelerated spring development of cheatgrass ( *Bromus tectorum* ) relative to native sagebrush vegetation, where warming and precipitation pulses advance flowering, with some latitudinal variation (Bradley 2009; Chambers et al. 2007; Howell et al. 2020). Early flowering allows invasive annuals to capitalize on early season resource windows, reducing direct competition with natives in spring but potentially increasing overlap in fall, when drought stress intensifies and phenology becomes more responsive to short‐term weather cues (Wolkovich and Cleland 2014; Wilsey et al. 2011). Cheatgrass in particular gains a competitive advantage in sagebrush habitats where summer drought suppresses native productivity (Brummer et al. 2016). Additional trait advantages, including higher nutrient‐use efficiency and longer leaf lifespans, may further enhance non‐native performance (Heberling and Fridley 2013; Wolkovich and Cleland 2014). Because phenology mediates competitive interactions and community structure, monitoring divergence at ecotones and native‐non‐native boundaries will be increasingly important under continued warming.

### 4.3 Temporal Variation and Climate

At the community‐level, flowering was stable across years but showed strong seasonal differences, further reflecting the bimodal growth strategy of the Great Basin Desert flora (Snyder et al. 2016; Ogle and Reynolds 2004). Across seasons, the spring flowering community had a greater richness and more advanced phenological stages than in fall. This observation is consistent with increasingly limited surface and ground water supply later in the growing season (Reynolds et al. 2004; Comstock and Ehleringer 1992; Crimmins et al. 2010; Bowers and Dimmitt 1994). These seasonal contrasts were further shaped by functional traits, with spring communities containing proportionally more annuals, herbaceous species, and non‐native taxa than fall.

As in other arid systems, precipitation timing and magnitude govern flowering predictability (Beatley 1974; Ogle and Reynolds 2004). Rainfall pulses generate strong seasonal and interannual variability, and the three‐fold higher spring richness and greater phenological consistency in our system are consistent with the broader ecological importance of moisture availability (Beatley 1974). Interannual differences in seasonal weather conditions preceding surveys were also evident during the study period, with cool, wet seasons generally associated with more advanced community flowering stages and warm, dry seasons corresponding with delayed development. In arid ecosystems, precipitation pulses and soil moisture recharge often exert stronger control over plant development than thermal accumulation, influencing plant germination, growth, and reproductive timing (Beatley 1974; Ogle and Reynolds 2004). The markedly high snowpack and precipitation observed in 2023 likely enhanced moisture availability during early spring, potentially contributing to early community flowering that survey year. Because fall surveys occurred just after absolute peak flowering, this confounds the ability to detect weather‐linked patterns. However, the fall flowering community may be more responsive to abiotic cues such as photoperiod (Wolkovich and Cleland 2014).

Exploratory mixed‐effects modeling suggested weak associations between climatic conditions and spring community flowering phenology. Among candidate models, although precipitation ranked marginally higher than other predictors, no climate variable received strong support, indicating limited explanatory power of the climatic variables considered. Temperature‐derived metrics, including chilling, forcing accumulation, and freezing exposure, received comparatively little support. Despite the weak statistical separation among models, the relative ranking of precipitation is consistent with the ecological importance of moisture availability in arid ecosystems, where precipitation strongly influences germination, growth, and reproductive timing (Beatley 1974; Ogle and Reynolds 2004). Given the limited temporal replication of four survey years and the comparable support for the null model, these relationships should be interpreted as preliminary patterns requiring confirmation through longer‐term monitoring capable of resolving persistent climate–phenology coupling across broader climatic conditions. Because species functional traits varied significantly among phenological stages, longer‐term datasets may additionally reveal trait‐mediated phenological responses that are obscured in these exploratory community‐level climate–phenology analyses.

The Great Basin Desert is an asynchronous hotspot where dynamic precipitation regimes, topography, and community composition drive fine‐scale temporal mosaics (Terasaki Hart et al. 2025). In Reno, the locally warming climate is projected to warm by 4.1°C–5.7°C (7.3°F–10.3°F) and become 18%–26.3% wetter in summer and fall, respectively (Rafferty et al. 2020; Hall and Willis 2006; Fitzpatrick and Dunn 2019). As the Reno region warms among the fastest in the United States (Fitzpatrick and Dunn 2019; Climate Central 2024), emerging phenological shifts have heightened ecological consequences. Species closely tracking climate cues show reduced long‐term declines (Wolkovich et al. 2013; Wainwright et al. 2012; Wolkovich and Cleland 2014), whereas low‐plasticity species may be more vulnerable (Anderson et al. 2012; Ghalambor et al. 2007). Even modest phenological shifts can alter pollinator networks (Forrest and Thomson 2011; Kudo and Cooper 2019), gene flow (Hall and Willis 2006), and competition (Wolkovich and Cleland 2014). Continued monitoring will be essential for detecting early signs of temporal asynchrony and flowering community stability in these rapidly warming systems.

### 4.4 Value of Rapid Phenology Surveys in Arid Ecosystems

Traditional phenology studies often rely on repeated observations or early season firsts, which can inflate interannual variability and obscure community‐level patterns (CaraDonna et al. 2014). Because floral stages directly reflect reproductive opportunity and pollinator resources, assessing community status at peak bloom provides a robust metric of phenological structure (Martins et al. 2021; Denny et al. 2014; Kudo and Cooper 2019). Our findings support rapid, peak‐season surveys as an effective method for arid systems where flowering is episodic, spatially heterogeneous, and continuous monitoring is challenging (Wolkovich et al. 2013; Rafferty et al. 2020).

The five‐stage floral classification offers a rapid but coarse measure of phenology; finer categorical scales or inclusion of floral abundance may reveal subtler within‐season differences (Denny et al. 2014; Katal et al. 2022). Fall surveys occurred shortly after absolute peak flowering, and alongside a lower community diversity and one fewer sampling season than in spring, reduced the power of seasonal comparisons. The multi‐year record is also too short to separate climate‐driven trends from annual variability, a common constraint in arid phenology research (Liancourt et al. 2012). Because analyses were based on four annual observations, statistical assumptions underlying correlation, regression, and model‐selection approaches could not be rigorously evaluated. Accordingly, analyses were interpreted descriptively to identify consistent directional patterns rather than to support formal statistical inference. Consequently, the emergent but statistically weak climate–phenology emerging from mixed‐effects model comparisons reflects limited temporal scope rather than weak ecological coupling, underscoring the need for longer‐term monitoring (Iler et al. 2017).

Incorporating abundance data could further clarify the dynamics of demographic responses and community interactions, though the stabilizing effect of diversity on phenology may be independent of abundance (CaraDonna et al. 2014; Dorado and Vázquez 2016). The underlying mechanisms of phenotypic plasticity, genetic differentiation, or phylogenetic constraints remain a key challenge for predicting phenological responses to climate change. Although longer‐term datasets are needed to disentangle these mechanisms, rapid community‐level surveys such as ours provide an essential foundation for detecting early phenological shifts in arid ecosystems.

### 4.5 Conclusions

Our rapid‐survey protocol effectively quantified community flowering phenology in an arid plant community, revealing temporal and functional patterns consistent with interannual climate variability. In one of North America's fastest‐warming regions, exploratory mixed‐effects models indicated limited support for climate predictors of community flowering phenology, highlighting the need for longer time series to resolve climate–phenology relationships. Rapid community‐level phenology monitoring provides a practical approach for arid, climate‐sensitive systems where resources for intensive monitoring are limited, offering a foundation for detecting phenological change and guiding long‐term monitoring efforts in rapidly changing arid ecosystems.

## Author Contributions

**Megan E. Lahti:** conceptualization (equal), data curation (equal), formal analysis (supporting), funding acquisition (supporting), investigation (lead), methodology (equal), validation (supporting), visualization (supporting), writing – original draft (lead), writing – review and editing (lead). **Eriko Sakamura:** data curation (equal), formal analysis (equal), validation (equal), visualization (equal), writing – original draft (supporting), writing – review and editing (supporting). **Minsung Jung:** data curation (equal), formal analysis (equal), validation (equal), visualization (supporting), writing – original draft (supporting), writing – review and editing (supporting). **Cecilia M. Vigil:** conceptualization (equal), data curation (supporting), formal analysis (supporting), funding acquisition (lead), investigation (supporting), methodology (equal), writing – original draft (supporting), writing – review and editing (supporting). **Kiley S. Smith:** data curation (supporting), formal analysis (supporting), visualization (supporting), writing – original draft (supporting), writing – review and editing (supporting). **Megan Ramirez:** data curation (supporting), formal analysis (supporting), visualization (supporting), writing – original draft (supporting), writing – review and editing (supporting). **Alec C. Brooks:** data curation (supporting), formal analysis (supporting), visualization (supporting), writing – original draft (supporting), writing – review and editing (supporting).

## Funding

This work was supported by the National Institute of General Medical Sciences of the National Institute of Health, GM103440.

## Conflicts of Interest

The authors declare no conflicts of interest.

## Supporting information


**Data S1:** R code used for statistical analyses.

**Table S1:** Species‐level phenological observations and functional trait data across years, seasons, and transects in a Great Basin Desert plant community. This table includes all plant observations used in analyses of seasonal phenology from 2021 to 2024 across spring and fall surveys. Each record represents a species occurrence within a transect and survey period, along with its modal phenological stage. Functional traits include growth form (forb, shrub), nativity status (native, non‐native), and lifespan (annual herbaceous, perennial herbaceous, perennial woody). Phenological status was recorded using binary indicators for reproductive stages (only buds, early flowering, peak flowering, late flowering, only fruits), where 1 indicates presence and 0 indicates absence during a survey. A continuous phenology index (range: 1–5) was calculated to represent sequential reproductive stages, where 1 = buds only, 2 = early flowering, 3 = peak flowering, 4 = late flowering, 5 = fruiting (flowers absent). Phenology values were assigned as the modal stage across ≥ 30 individuals per species; when two adjacent stages were equally frequent, transitional values were recorded as the mean of those stages (0.5 increments).

## Data Availability

Phenological data and R code for the analyses are provided as Data S1. Functional trait data used in this analysis and presented in Table S1 are available through The Biota of North America Program (BONAP) Taxonomic Data Center web portal (http://www.bonap.net/tdc). Climate data used in this analysis for temperature and precipitation are available through the National Oceanic and Atmospheric Administration National Centers for Environmental Information Global Historical Climatology Network daily web portal for the Reno Airport (USW00023185) station (https://www.ncei.noaa.gov/) and for snow water content are available through the California Department of Water Resources California Data Exchange Center web portal the Big Meadows (BMW) station (https://cdec.water.ca.gov/snowapp/sweq.action). Survey plot physical characteristics summary data from Table 1 are available through the United States Department of Agriculture Natural Resources Conservation Service Web Soil Survey web portal (https://websoilsurvey.sc.egov.usda.gov/). The Figure 1 feature layer containing 2 ft. elevation contour lines is available for download from City of Reno GIS Open Data Hub at https://data‐cityofreno.opendata.arcgis.com.

## References

[ece373340-bib-0001] Amano, T. , R. J. Smithers , T. H. Sparks , and W. J. Sutherland . 2010. “A 250‐Year Index of First Flowering Dates and Its Response to Temperature Changes.” Proceedings of the Royal Society B: Biological Sciences 277, no. 1693: 2451–2457. 10.1098/rspb.2010.0291. PMC2894925 20375052

[ece373340-bib-0002] Anderson, J. T. , D. W. Inouye , A. M. McKinney , R. I. Colautti , and T. Mitchell‐Olds . 2012. “Phenotypic Plasticity and Adaptive Evolution Contribute to Advancing Flowering Phenology in Response to Climate Change.” Proceedings of the Royal Society B: Biological Sciences 279: 3843–3852. 10.1098/rspb.2012.1051. PMC3415914 22787021

[ece373340-bib-0003] Balmaki, B. , M. A. Rostami , J. M. Allen , and L. A. Dyer . 2024. “Effects of Climate Change on Lepidoptera Pollen Loads and Their Pollination Services in Space and Time.” Oecologia 204: 751–759. 10.1007/s00442-024-05533-y. 38523192

[ece373340-bib-0004] Bates, D. , M. Mächler , B. Bolker , and S. Walker . 2015. “Fitting Linear Mixed‐Effects Models Using lme4.” Journal of Statistical Software 67: 1–48. 10.18637/jss.v067.i01.

[ece373340-bib-0005] Beatley, J. C. 1974. “Phenological Events and Their Environmental Triggers in Mojave Desert Ecosystems.” Ecology 55, no. 4: 856–863. 10.2307/1934421.

[ece373340-bib-0006] Bennett, A. F. , D. G. Nimmo , and J. Q. Radford . 2014. “Riparian Vegetation Has Disproportionate Benefits for Landscape‐Scale Conservation of Woodland Birds in Highly Modified Environments.” Journal of Applied Ecology 51: 514–523. 10.1111/1365-2664.12200.

[ece373340-bib-0007] Bowers, J. E. , and M. A. Dimmitt . 1994. “Flowering Phenology of Six Woody Plants in the Northern Sonoran Desert.” Bulletin of the Torrey Botanical Club 121, no. 3: 215. 10.2307/2997177.

[ece373340-bib-0008] Bradley, B. A. 2009. “Regional Analysis of the Impacts of Climate Change on Cheatgrass Invasion Shows Potential Risk and Opportunity.” Global Change Biology 15, no. 1: 196–208. 10.1111/j.1365-2486.2008.01709.x.

[ece373340-bib-0009] Brummer, T. J. , K. T. Taylor , J. Rotella , B. D. Maxwell , L. J. Rew , and M. Lavin . 2016. “Drivers of *Bromus tectorum* Abundance in the Western North American Sagebrush Steppe.” Ecosystems 19: 986–1000. 10.1007/s10021-016-9980-3.

[ece373340-bib-0078] California Department of Water Resources . 2024. “California Data Exchange Center.” https://cdec.water.ca.gov/.

[ece373340-bib-0010] CaraDonna, P. J. , A. M. Iler , and D. W. Inouye . 2014. “Shifts in Flowering Phenology Reshape a Subalpine Plant Community.” Proceedings of the National Academy of Sciences 111, no. 13: 4916–4921. 10.1073/pnas.1323073111. PMC3977233 24639544

[ece373340-bib-0011] Chambers, J. C. , B. A. Roundy , R. R. Blank , S. E. Meyer , and A. Whittaker . 2007. “What Makes Great Basin Sagebrush Ecosystems Invasible by *Bromus tectorum* ?” Ecological Monographs 77, no. 1: 117–145. 10.1890/05-1991.

[ece373340-bib-0012] Chesson, P. 2000. “Mechanisms of Maintenance of Species Diversity.” Annual Review of Ecology and Systematics 31, no. 1: 343–366. 10.1146/annurev.ecolsys.31.1.343.

[ece373340-bib-0013] Climate Central . 2023. Seasonal Allergies: Pollen and Mold Report 2023. Climate Central.

[ece373340-bib-0014] Climate Central . 2024. Earth Day: Fastest‐Warming Cities and Record Clean Investment Report 2024. Climate Central.

[ece373340-bib-0015] Comstock, J. P. , and J. R. Ehleringer . 1992. “Plant Adaptation in the Great Basin and Colorado Plateau.” Great Basin Naturalist 1: 195–215.

[ece373340-bib-0016] Cortés‐Flores, J. , K. B. Hernández‐Esquivel , A. González‐Rodríguez , and G. Ibarra‐Manríquez . 2017. “Flowering Phenology, Growth Forms, and Pollination Syndromes in Tropical Dry Forest Species: Influence of Phylogeny and Abiotic Factors.” American Journal of Botany 104: 39–49. 10.3732/ajb.1600305. 28031168

[ece373340-bib-0017] Crimmins, T. M. , M. A. Crimmins , and C. D. Bertelsen . 2010. “Complex Responses to Climate Drivers in Onset of Spring Flowering Across a Semi‐Arid Elevation Gradient.” Journal of Ecology 98: 1042–1051. 10.1111/j.1365-2745.2010.01696.x.

[ece373340-bib-0018] Davies, T. J. , E. M. Wolkovich , N. J. B. Kraft , et al. 2013. “Phylogenetic Conservatism in Plant Phenology.” Journal of Ecology 101: 1520–1530. 10.1111/1365-2745.12154.

[ece373340-bib-0019] Davis, C. C. , C. G. Willis , R. B. Primack , and A. J. Miller‐Rushing . 2010. “The Importance of Phylogeny to the Study of Phenological Response to Global Climate Change.” Philosophical Transactions of the Royal Society B 365, no. 1555: 3201–3213. 10.1098/rstb.2010.0130. PMC2981945 20819813

[ece373340-bib-0020] Denny, E. G. , K. L. Gerst , A. J. Miller‐Rushing , et al. 2014. “Standardized Phenology Monitoring Methods to Track Plant and Animal Activity for Science and Resource Management Applications.” International Journal of Biometeorology 58, no. 4: 591–601. 10.1007/s00484-014-0789-5. 24458770 PMC4023011

[ece373340-bib-0021] Dorado, J. , and D. P. Vázquez . 2016. “Flower Diversity and Bee Reproduction in an Arid Ecosystem.” PeerJ 4: e2250. 10.7717/peerj.2250. 27547556 PMC4974926

[ece373340-bib-0022] Elzinga, J. A. , A. Atlan , A. Biere , L. Gigord , A. E. Weis , and G. Bernasconi . 2007. “Time After Time: Flowering Phenology and Biotic Interactions.” Trends in Ecology & Evolution 22, no. 8: 432–439. 10.1016/j.tree.2007.05.006. 17573151

[ece373340-bib-0023] ESRI . 2025. ArcGIS Pro (Version 3.5.2) [Software]. ESRI Inc.

[ece373340-bib-0024] Fitzpatrick, M. C. , and R. R. Dunn . 2019. “Contemporary Climatic Analogs for 540 North American Urban Areas in the Late 21st Century.” Nature Communications 10, no. 1: 614. 10.1038/s41467-019-08540-3. PMC6372656 30755612

[ece373340-bib-0025] Forrest, J. , D. W. Inouye , and J. D. Thomson . 2010. “Flowering Phenology in Subalpine Meadows: Does Climate Variation Influence Community Co‐Flowering Patterns?” Ecology 91: 431–440. 10.1890/09-0099.1. 20392008

[ece373340-bib-0026] Forrest, J. R. K. 2015. “Plant–Pollinator Interactions and Phenological Change: What Can We Learn About Climate Impacts From Experiments and Observations?” Oikos 124, no. 1: 4–13. 10.1111/oik.01386.

[ece373340-bib-0027] Forrest, J. R. K. , and J. D. Thomson . 2011. “An Examination of Synchrony Between Insect Emergence and Flowering in Rocky Mountain Meadows.” Ecological Monographs 81: 469–491. 10.1890/10-1885.1.

[ece373340-bib-0028] Ghalambor, C. K. , J. K. McKay , S. P. Carroll , and D. N. Reznick . 2007. “Adaptive Versus Non‐Adaptive Phenotypic Plasticity and the Potential for Contemporary Adaptation in New Environments.” Functional Ecology 21: 394–407. 10.1111/j.1365-2435.2007.01283.x.

[ece373340-bib-0029] Hall, M. C. , and J. H. Willis . 2006. “Divergent Selection on Flowering Time Contributes to Local Adaptation in *Mimulus* Populations.” Evolution 60: 2466–2477. 10.1111/j.0014-3820.2006.tb01882.x. 17263109

[ece373340-bib-0030] Heberling, J. M. , and J. D. Fridley . 2013. “Resource‐Use Strategies of Native and Invasive Plants in Eastern North American Forests.” New Phytologist 200: 523–533. 10.1111/nph.12388. 23815090

[ece373340-bib-0031] Howell, A. , D. E. Winkler , M. L. Phillips , B. McNellis , and S. C. Reed . 2020. “Experimental Warming Changes Phenology and Shortens Growing Season of the Dominant Invasive Plant *Bromus tectorum* (Cheatgrass).” Frontiers in Plant Science 11: 570001. 10.3389/fpls.2020.570001. 33178240 PMC7593257

[ece373340-bib-0032] Iler, A. M. , D. W. Inouye , N. M. Schmidt , and T. T. Høye . 2017. “Detrending Phenological Time Series Improves Climate–Phenology Analyses and Reveals Evidence of Plasticity.” Ecology 98: 647–655. 10.1002/ecy.1690. 27984645

[ece373340-bib-0033] Jiang, Y. , S. J. Mayor , X. Chu , et al. 2025. “Assessing Plant Phenological Changes Based on Drivers of Spring Phenology.” eLife 14: RP106655. 10.7554/eLife.106655.1. 41217818 PMC12604855

[ece373340-bib-0034] Kartesz, J. T. 2015. The Biota of North America Program (BONAP): Taxonomic Data Center. BONAP.

[ece373340-bib-0035] Katal, N. , M. Rzanny , P. Mäder , and J. Wäldchen . 2022. “Deep Learning in Plant Phenological Research: A Systematic Literature Review.” Frontiers in Plant Science 13: 805738. 10.3389/fpls.2022.805738. 35371160 PMC8969581

[ece373340-bib-0036] Klimešová, J. , M. P. Nobis , and T. Herben . 2016. “Links Between Shoot and Plant Longevity and Plant Economics Spectrum: Environmental and Demographic Implications.” Perspectives in Plant Ecology, Evolution and Systematics 22: 55–62. 10.1016/j.ppees.2016.09.002.

[ece373340-bib-0037] Körner, C. , and D. Basler . 2010. “Phenology Under Global Warming.” Science 327: 1461–1462. 10.1126/science.1186473. 20299580

[ece373340-bib-0038] Kudo, G. , and E. J. Cooper . 2019. “When Spring Ephemerals Fail to Meet Pollinators: Mechanism of Phenological Mismatch and Its Impact on Plant Reproduction.” Proceedings of the Royal Society B: Biological Sciences 286, no. 1904: 20190573. 10.1098/rspb.2019.0573. PMC6571468 31185863

[ece373340-bib-0039] LandFire . 2025. Existing Vegetation Type Layer, LANDFIRE 2.0.0 [Internet]. U.S. Department of the Interior, U.S. Geological Survey, & U.S. Department of Agriculture.

[ece373340-bib-0040] Lavorel, S. , and E. Garnier . 2002. “Predicting Changes in Community Composition and Ecosystem Functioning From Plant Traits: Revisiting the Holy Grail.” Functional Ecology 16: 545–556. 10.1046/j.1365-2435.2002.00664.x.

[ece373340-bib-0041] Liancourt, P. , L. A. Spence , B. Boldgiv , et al. 2012. “Vulnerability of the Northern Mongolian Steppe to Climate Change: Insights From Flower Production and Phenology.” Ecology 93, no. 4: 815–824. 10.1890/11-1003.1. 22690632

[ece373340-bib-0042] López López, J. I. , V. Parra Tabla , and D. Mondragón . 2020. “Variation in the Flowering Phenology of an Epiphytic Bromeliad Along an Elevational Gradient.” Acta Biologica Colombiana 26, no. 1: 42–53. 10.15446/abc.v26n1.82875.

[ece373340-bib-0043] Luo, Z. , O. J. Sun , Q. Ge , W. Xu , and J. Zheng . 2007. “Phenological Responses of Plants to Climate Change in an Urban Environment.” Ecological Research 22, no. 3: 507–514. 10.1007/s11284-006-0044-6.

[ece373340-bib-0044] Martins, A. E. , M. G. G. Camargo , and L. P. C. Morellato . 2021. “Flowering Phenology and the Influence of Seasonality in Flower Conspicuousness for Bees.” Frontiers in Plant Science 11: 594538. 10.3389/fpls.2020.594538. 33664750 PMC7921784

[ece373340-bib-0045] Matthews, E. R. , and S. J. Mazer . 2015. “Historical Changes in Flowering Phenology Are Governed by Temperature × Precipitation Interactions in a Widespread Perennial Herb in Western North America.” New Phytologist 210, no. 1: 157–167. 10.1111/nph.13751. 26595165

[ece373340-bib-0046] Menzel, A. , T. H. Sparks , N. Estrella , et al. 2006. “European Phenological Response to Climate Change Matches the Warming Pattern.” Global Change Biology 12, no. 10: 1969–1976. 10.1111/j.1365-2486.2006.01193.x.

[ece373340-bib-0079] National Interagency Fire Center . 2025. “NIFC Open Data Site.” https://data‐nifc.opendata.arcgis.com/.

[ece373340-bib-0047] National Oceanic and Atmospheric Administration . 2024. “National Centers for Environmental Information.” https://www.ncei.noaa.gov.

[ece373340-bib-0048] Ogle, K. , and J. F. Reynolds . 2004. “Plant Responses to Precipitation in Desert Ecosystems: Integrating Functional Types, Pulses, Thresholds, and Delays.” Oecologia 141, no. 2: 282–294. 10.1007/s00442-004-1507-5. 15007725

[ece373340-bib-0049] Oksanen, J. , G. Simpson , F. Blanchet , et al. 2025. “Vegan: Community Ecology Package, Version 2.7‐2.” https://10.32614/CRAN.package.vegan.

[ece373340-bib-0050] Pareja‐Bonilla, D. , M. Arista , L. P. C. Morellato , and P. L. Ortiz . 2025. “Better Soon Than Never: Climate Change Induces Strong Phenological Reassembly in the Flowering of a Mediterranean Shrub Community.” Annals of Botany 135, no. 1–2: 239–254. 38099507 10.1093/aob/mcad193 PMC11805945

[ece373340-bib-0051] Park, J. S. , and E. Post . 2022. “Seasonal Timing on a Cyclical Earth: Towards a Theoretical Framework for the Evolution of Phenology.” PLoS Biology 20, no. 12: e3001952. 10.1371/journal.pbio.3001952. 36574457 PMC9829184

[ece373340-bib-0052] Parmesean, C. 2007. “Influences of Species, Latitudes and Methodologies on Estimates of Phenological Response to Global Warming.” Global Change Biology 13: 1860–1872. 10.1111/j.1365-2486.2007.01404.x.

[ece373340-bib-0053] Peel, M. C. , B. L. Finlayson , and T. A. McMahon . 2007. “Updated World Map of the Köppen‐Geiger Climate Classification.” Hydrology and Earth System Sciences 11, no. 5: 1633–1644. 10.5194/hess-11-1633-2007.

[ece373340-bib-0054] Peterson, E. B. 2008. A Synthesis of Vegetation Maps for Nevada (Initiating a ‘Living’ Vegetation Map). Nevada Natural Heritage Program Documentation and geospatial data.

[ece373340-bib-0055] Pohlert, T. 2024. PMCMRplus: Calculate Pairwise Multiple Comparisons of Mean Rank Sums Extended. Version 1.9.12. R Foundation for Statistical Computing.

[ece373340-bib-0056] Poole, J. A. , C. S. Barnes , J. G. Demain , et al. 2019. “Impact of Weather and Climate Change With Indoor and Outdoor Air Quality in Asthma: A Work Group Report of the AAAAI Environmental Exposure and Respiratory Health Committee.” Journal of Allergy and Clinical Immunology 143, no. 5: 1702–1710. 10.1016/j.jaci.2019.02.018. 30826366 PMC10907958

[ece373340-bib-0057] Primack, R. B. , H. Higuchi , and A. J. Miller‐Rushing . 2009. “The Impact of Climate Change on Cherry Trees and Other Species in Japan.” Biological Conservation 142, no. 9: 1943–1949. 10.1016/j.biocon.2009.03.016.

[ece373340-bib-0058] R Core Team . 2024. R: A Language and Environment for Statistical Computing. R Foundation for Statistical Computing.

[ece373340-bib-0059] Rafferty, N. E. , J. M. Diez , and C. D. Bertelsen . 2020. “Changing Climate Drives Divergent and Nonlinear Shifts in Flowering Phenology Across Elevations.” Current Biology 30, no. 3: 432–441. 10.1016/j.cub.2019.11.071. 31902725

[ece373340-bib-0060] Rathcke, B. , and E. P. Lacey . 1985. “Phenological Patterns of Terrestrial Plants.” Annual Review of Ecology and Systematics 16: 179–214. 10.1146/annurev.es.16.110185.001143.

[ece373340-bib-0061] Reynolds, J. F. , P. R. Kemp , K. Ogle , and R. J. Fernández . 2004. “Modifying the ‘Pulse–Reserve’ Paradigm for Deserts of North America: Precipitation Pulses, Soil Water, and Plant Responses.” Oecologia 141: 194–210. 10.1007/s00442-004-1524-4. 15042457

[ece373340-bib-0062] Richardson, A. D. , T. A. Black , P. Ciais , et al. 2010. “Influence of Spring and Autumn Phenological Transitions on Forest Ecosystem Productivity.” Philosophical Transactions of the Royal Society of London. Series B, Biological Sciences 365, no. 1555: 3227–3246. 10.1098/rstb.2010.0102. 20819815 PMC2981939

[ece373340-bib-0063] Salguero‐Gómez, R. , O. R. Jones , E. Jongejans , et al. 2016. “Fast–Slow Continuum and Reproductive Strategies Structure Plant Life‐History Variation Worldwide.” Proceedings of the National Academy of Sciences 113, no. 1: 230–235. 10.1073/pnas.1506215112. PMC4711876 26699477

[ece373340-bib-0064] Shaw, J. R. , and D. J. Cooper . 2008. “Linkages Among Watersheds, Stream Reaches, and Riparian Vegetation in Dryland Ephemeral Stream Networks.” Journal of Hydrology 350, no. 1–2: 68–82. 10.1016/j.jhydrol.2007.11.030.

[ece373340-bib-0065] Sherry, R. A. , X. Zhou , S. Gu , et al. 2007. “Divergence of Reproductive Phenology Under Climate Warming.” Proceedings of the National Academy of Sciences of the United States of America 104, no. 1: 198–202. 10.1073/pnas.0605642104. 17182748 PMC1713188

[ece373340-bib-0066] Snyder, K. A. , B. L. Wehan , G. Filippa , J. L. Huntington , T. K. Stringham , and D. K. Snyder . 2016. “Extracting Plant Phenology Metrics in a Great Basin Watershed: Methods and Considerations for Quantifying Phenophases in a Cold Desert.” Sensors (Basel, Switzerland) 16, no. 11: 1948. 10.3390/s16111948. 27869752 PMC5134607

[ece373340-bib-0067] Song, C. , L. Zhang , Y. Jia , and D. Wu . 2025. “Phylogenetic Relatedness and Plant Traits Influenced Flowering Phenology Change Patterns in Natural Habitats in China (2003–2021).” BMC Plant Biology 25, no. 1: 259. 10.1186/s12870-025-06572-0. 40382560 PMC12085019

[ece373340-bib-0068] Terasaki Hart, D. E. , T.‐N. Bùi , L. Di Maggio , and I. J. Wang . 2025. “Global Phenology Maps Reveal the Drivers and Effects of Seasonal Asynchrony.” Nature 645: 133–140. 10.1038/s41586-025-09410-3. 40866701 PMC12408380

[ece373340-bib-0069] Wainwright, C. E. , E. M. Wolkovich , and E. E. Cleland . 2012. “Seasonal Priority Effects: Implications for Invasion and Restoration in a Semi‐Arid System.” Journal of Applied Ecology 49: 234–241. 10.1111/j.1365-2664.2011.02088.x.

[ece373340-bib-0070] Wan, S. , T. Yuan , S. Bowdish , L. Wallace , S. D. Russell , and Y. Luo . 2002. “Response of an Allergenic Species, *Ambrosia psilostachya* (Asteraceae), to Experimental Warming and Clipping: Implications for Public Health.” American Journal of Botany 89, no. 11: 1843–1846. 10.3732/ajb.89.11.1843. 21665612

[ece373340-bib-0071] Washoe County . 2025. Rancho San Rafael History. Washoe County.

[ece373340-bib-0072] Willis, C. G. , B. R. Ruhfel , R. B. Primack , A. J. Miller‐Rushing , J. B. Losos , and C. C. Davis . 2010. “Favorable Climate Change Response Explains Non‐Native Species' Success in Thoreau's Woods.” PLoS One 5, no. 1: e8878. 10.1371/journal.pone.0008878. 20126652 PMC2811191

[ece373340-bib-0073] Wilsey, B. J. , P. P. Daneshgar , and H. W. Polley . 2011. “Biodiversity, Phenology and Temporal Niche Differences Between Native‐ and Novel Exotic‐Dominated Grasslands.” Perspectives in Plant Ecology, Evolution and Systematics 13, no. 4: 265–276. 10.1016/j.ppees.2011.07.002.

[ece373340-bib-0074] Wolf, A. A. , E. S. Zavaleta , and P. C. Selmants . 2017. “Flowering Phenology Shifts in Response to Biodiversity Loss.” Proceedings of the National Academy of Sciences 114, no. 13: 3463–3468. 10.1073/pnas.1608357114. PMC5380019 28289231

[ece373340-bib-0075] Wolkovich, E. M. , and E. E. Cleland . 2014. “Phenological Niches and the Future of Invaded Ecosystems With Climate Change.” AoB Plants 6: plu013. 10.1093/aobpla/plu013. 24876295 PMC4025191

[ece373340-bib-0076] Wolkovich, E. M. , T. J. Davies , H. Schaefer , et al. 2013. “Temperature‐Dependent Shifts in Phenology Contribute to the Success of Exotic Species With Climate Change.” American Journal of Botany 100, no. 7: 1407–1421. 10.3732/ajb.1200478. 23797366

[ece373340-bib-0077] Zhang, X. , M. A. Friedl , C. B. Schaaf , and A. H. Strahler . 2004. “Climate Controls on Vegetation Phenological Patterns in Northern Mid‐ and High Latitudes Inferred From MODIS Data.” Global Change Biology 10, no. 7: 1133–1145. 10.1111/j.1365-2486.2004.00784.x.

