# Supplementary material for: Rapid Surveys Reveal Temporal Variation in Flowering Community Phenology in a Great Basin Desert Ecosystem
Source: Ecol Evol. 2026 Apr 1;16(4):e73340. doi: 10.1002/ece3.73340 (PMC13045367; doi:10.1002/ece3.73340)
Supplement: Supplementary file 1 — Data S1: R code used for statistical analyses. [file ECE3-16-e73340-s002.pdf]

R Code Used for Statistical Analyses

Manuscript Title:
Rapid Surveys Reveal Temporal Variation in Flowering-Community Phenology in a Great Basin Desert Ecosystem

Authors:
Megan E. Lahti1* (corresponding; mlahti@tmcc.edu), Eriko Sakamura,2, Minsung Jung3, Cecilia M. Vigil1, Kiley S. Smith1, Megan Ramirerz1, Alec C. Brooks 2

Description:
This document contains the R scripts used for statistical analyses associated with this study. All analyses were conducted in **R version 4.4.2.**

Species Effort Curve (SEC)

```
library(dplyr)
library(tidyr)
library(vegan)
library(ggplot2)
library(readxl)

df <- read_excel("Clean_Flr_Data_Edit_MEL.xlsx")

# sample id
df$sample_id <- paste(df$Transect, df$season, df$Year, sep = "_")

# all samples
comm <- group_by(df, sample_id, Species)
comm <- summarize(comm, present = 1L, .groups = "drop")
comm <- pivot_wider(
  comm,
  names_from = Species,
  values_from = present,
  values_fill = 0
)
comm <- arrange(comm, sample_id)

sample_order <- comm$sample_id
comm2 <- as.data.frame(comm[, setdiff(names(comm), "sample_id"), drop = FALSE])

env <- distinct(df, sample_id, Transect, season, Year)
env <- arrange(env, factor(sample_id, levels = sample_order))
```

```
set.seed(2025)
sec_model <- specaccum(comm2, method = "random", permutations = 1000)

sec_df <- data.frame(
  Samples = sec_model$sites,
  Richness = sec_model$richness,
  SD = sec_model$sd
)

ggplot(sec_df, aes(x = Samples, y = Richness)) +
  geom_line(color = "#1f78b4", linewidth = 1.2) +
  geom_ribbon(
    aes(ymin = Richness - SD, ymax = Richness + SD),
    fill = "#1f78b4",
    alpha = 0.2
  ) +
  labs(
    title = "SEC — All Samples",
    x = "Number of Samples",
    y = "Species Richness"
  ) +
  theme_minimal(base_size = 14)

# by season
df$sample_id <- paste(df$Transect, df$season, df$Year, sep = "_")

comm <- group_by(df, sample_id, season, Species)
comm <- summarize(comm, present = 1L, .groups = "drop")
comm <- pivot_wider(
```

```
  comm,
  names_from = Species,
  values_from = present,
  values_fill = 0
)
comm <- arrange(comm, season, sample_id)

comm_list <- split(comm, comm$season)

set.seed(2025)
sec_results <- lapply(names(comm_list), function(season_name) {
  temp <- comm_list[[season_name]]
  temp <- select(temp, -sample_id, -season)

  if (nrow(temp) > 1) {
    model <- specaccum(temp, method = "random", permutations = 500)

    df_out <- data.frame(
      Season = season_name,
      Samples = model$sites,
      Richness = model$richness,
      SD = model$sd
    )

    df_out <- rbind(
      data.frame(Season = season_name, Samples = 0, Richness = 0, SD = 0),
      df_out
    )
```

```
    df_out
  } else {
    NULL
  }
})

sec_df_season <- bind_rows(sec_results)

ggplot(sec_df_season, aes(x = Samples, y = Richness, color = Season, fill = Season)) +
  geom_line(linewidth = 1.2) +
  geom_ribbon(
    aes(ymin = Richness - SD, ymax = Richness + SD),
    alpha = 0.2,
    color = NA
  ) +
  scale_x_continuous(expand = expansion(mult = c(0, 0.05))) +
  labs(
    title = "SEC by Season",
    x = "Number of Samples",
    y = "Species Richness"
  ) +
  theme_minimal(base_size = 14) +
  theme(legend.position = "top")

ggplot(sec_df_season, aes(x = Samples, y = Richness, color = Season, fill = Season)) +
  geom_line(linewidth = 1.2) +
  geom_ribbon(
    aes(ymin = Richness - SD, ymax = Richness + SD),
    alpha = 0.2,
```

```
    color = NA
  ) +
  scale_color_manual(values = c(
    "Spring" = "SteelBlue2",
    "Fall" = "DarkGoldenrod"
  )) +
  scale_fill_manual(values = c(
    "Spring" = "SteelBlue2",
    "Fall" = "DarkGoldenrod"
  )) +
  scale_x_continuous(expand = expansion(mult = c(0, 0.05))) +
  labs(
    title = "SEC by Season",
    x = "Number of Samples",
    y = "Species Richness"
  ) +
  theme_minimal(base_size = 14) +
  theme(legend.position = "right")

# by transect
df$sample_id <- paste(df$Transect, df$season, df$Year, sep = "_")

comm <- group_by(df, sample_id, Transect, Species)
comm <- summarize(comm, present = 1L, .groups = "drop")
comm <- pivot_wider(
  comm,
  names_from = Species,
  values_from = present,
  values_fill = 0
```

```
)

comm_list <- split(comm, comm$Transect)

set.seed(2025)
sec_results <- lapply(names(comm_list), function(transect_name) {
  temp <- comm_list[[transect_name]]
  temp <- select(temp, -Transect, -sample_id)

  model <- specaccum(temp, method = "random", permutations = 1000)

  data.frame(
    Transect = transect_name,
    Samples = model$sites,
    Richness = model$richness,
    SD = model$sd
  )
})

sec_df <- bind_rows(sec_results)

ggplot(sec_df, aes(x = Samples, y = Richness, color = Transect, fill = Transect)) +
  geom_line(linewidth = 1.2) +
  geom_ribbon(
    aes(ymin = Richness - SD, ymax = Richness + SD),
    alpha = 0.2,
    color = NA
  ) +
  labs(
```

```
    title = "SEC by Transect (All Seasons)",
    x = "Number of Samples",
    y = "Species Richness"
  ) +
  theme_minimal(base_size = 14) +
  theme(legend.position = "top")

ggplot(sec_df, aes(x = Samples, y = Richness)) +
  geom_line(color = "#1f78b4", linewidth = 1.1) +
  geom_ribbon(
    aes(ymin = Richness - SD, ymax = Richness + SD),
    fill = "#1f78b4",
    alpha = 0.2
  ) +
  facet_wrap(~ Transect) +
  labs(
    title = "SEC by Transect (All Seasons)",
    x = "Number of Samples",
    y = "Species Richness"
  ) +
  theme_minimal(base_size = 14)

# by transect and season
df$sample_id <- paste(df$Transect, df$season, df$Year, sep = "_")

comm <- group_by(df, sample_id, Transect, season, Species)
comm <- summarize(comm, present = 1L, .groups = "drop")
comm <- pivot_wider(
  comm,
```

```
  names_from = Species,
  values_from = present,
  values_fill = 0
)

comm_list <- split(comm, list(comm$Transect, comm$season), drop = TRUE)

set.seed(2025)
sec_results <- lapply(names(comm_list), function(group_name) {
  temp <- comm_list[[group_name]]
  temp_comm <- select(temp, -Transect, -season, -sample_id)

  if (nrow(temp_comm) > 1) {
    model <- specaccum(temp_comm, method = "random", permutations = 1000)

    parts <- strsplit(group_name, "\\.")[[1]]
    transect <- parts[1]
    season <- parts[2]

    data.frame(
      Transect = transect,
      Season = season,
      Samples = model$sites,
      Richness = model$richness,
      SD = model$sd
    )
  } else {
    NULL
  }
```

```
})

sec_df <- bind_rows(sec_results)

ggplot(sec_df, aes(x = Samples, y = Richness, color = Season, fill = Season)) +
  geom_line(linewidth = 1.2) +
  geom_ribbon(
    aes(ymin = Richness - SD, ymax = Richness + SD),
    alpha = 0.2,
    color = NA
  ) +
  facet_wrap(~ Transect, scales = "free_x") +
  labs(
    title = "SEC by Transect and Season",
    x = "Number of Samples",
    y = "Species Richness"
  ) +
  theme_minimal(base_size = 14) +
  theme(
    legend.position = "top",
    strip.text = element_text(face = "bold")
  )

# by season and transect
df$sample_id <- paste(df$Transect, df$season, df$Year, sep = "_")

comm <- group_by(df, sample_id, Transect, season, Species)
comm <- summarize(comm, present = 1L, .groups = "drop")
comm <- pivot_wider(
```

```
  comm,
  names_from = Species,
  values_from = present,
  values_fill = 0
)

comm_list <- split(comm, list(comm$season, comm$Transect), drop = TRUE)

set.seed(2025)
sec_results <- lapply(names(comm_list), function(group_name) {
  temp <- comm_list[[group_name]]
  temp_comm <- select(temp, -sample_id, -Transect, -season)

  if (nrow(temp_comm) > 1) {
    model <- specaccum(temp_comm, method = "random", permutations = 1000)

    parts <- strsplit(group_name, "\\.")[[1]]
    season <- parts[1]
    transect <- parts[2]

    data.frame(
      Season = season,
      Transect = transect,
      Samples = model$sites,
      Richness = model$richness,
      SD = model$sd
    )
  } else {
    NULL
```

```
  }
})

sec_df <- bind_rows(sec_results)

ggplot(sec_df, aes(x = Samples, y = Richness, color = Transect, fill = Transect)) +
  geom_line(linewidth = 1.2) +
  geom_ribbon(
    aes(ymin = Richness - SD, ymax = Richness + SD),
    alpha = 0.2,
    color = NA
  ) +
  facet_wrap(~ Season, ncol = 1) +
  labs(
    title = "SEC by Season and Transect in Each Season",
    x = "Number of Samples",
    y = "Species Richness"
  ) +
  theme_minimal(base_size = 14) +
  theme(
    legend.position = "top",
    strip.text = element_text(face = "bold", size = 13)
  )
```

Seasonal Phenology: FriedmanPostHocSpring

```
#install.packages("PMCMRplus")
#install.packages("readr")
#install.packages("dplyr")
#install.packages("broom")
```

```r
library(readr)

library(PMCMRplus)

library(dplyr)

library(knitr)

library(broom)

df <- read_csv("../data/ecoblitz_spring.csv")

df_mean <- df %>%

  group_by(Transect, Year) %>%

  summarise(

    Phenology = mean(Phenology, na.rm = TRUE),

    .groups = "drop"

  )

#mean Table 1

rep_cells <- df_mean %>% mutate(Phenology = round(Phenology, 2))

kable(rep_cells, caption = "Aggregated Transect × Year Mean Phenology Values")

#friedmen and table 2

resF <- friedman.test(Phenology~ Year | Transect, data = df_mean)

friedman_table <- data.frame(

  `Chi-squared`

  = round(resF$statistic, 3),

  `df`

  = resF$parameter,

  `p-value`

  = signif(resF$p.value, 4)

)

kable(friedman_table, caption = "Friedman Test Results")

#Nemenyi and table 3

resH <- frdAllPairsNemenyiTest(Phenology~ Year | Transect, data = df_mean)

kable(round(resH$p.value, 4), caption = "Pairwise Nemenyi Test p-value Matrix")
```

```
#table 4
pv <- resH$p.value
pv_long <- as.data.frame(as.table(pv), stringsAsFactors = FALSE) %>%
  rename(row = Var1, col = Var2, p.value = Freq) %>%
  filter(!is.na(p.value))
hoc_table <- pv_long %>%
  mutate(
    Comparison = ifelse(row < col, paste(row, "vs", col), paste(col, "vs", row)),
    `p-value`
    = round(p.value, 3),
    Interpretation = ifelse(
      p.value < 0.10,
      paste("**Significant → phenology differs between", Comparison, "**"),
      "Not significant"
    )
  ) %>%
  distinct(Comparison, .keep_all = TRUE) %>%
  select(Comparison,`p-value`, Interpretation) %>%
  arrange(`p-value`)
kable(hoc_table, caption = "Pairwise Nemenyi Test Results with Interpretation")
```

Phenological Structure: PERMANOVA_withinTransectAllSeasons

```
library(dplyr)
library(tidyr)
library(vegan)
library(readr)
library(ggplot2)
library(readxl)
```

```
df <- read_excel("Clean_Flr_Data_Edit_MEL.xlsx")

# clean data
df_clean <- filter(df, !is.na(Phenology), !is.na(Year), !is.na(season), !is.na(Transect))

# distance
phenology_dist <- dist(df_clean$Phenology, method = "euclidean")

# permanova
permanova_repeated <- adonis2(
  phenology_dist ~ Year * season,
  data = df_clean,
  permutations = 999,
  strata = df_clean$Transect
)

print(permanova_repeated)

# pairwise function
pairwise_permanova <- function(values, groups, strata_factor, perm = 999, p.adjust.method =
"bonferroni") {
  groups <- factor(groups)
  pairs <- combn(levels(groups), 2, simplify = TRUE)
  results <- list()

  for (i in 1:ncol(pairs)) {
    pair <- pairs[, i]
    idx <- which(groups %in% pair)
    dist_sub <- dist(values[idx], method = "euclidean")
```

```
    strata_sub <- strata_factor[idx]
    group_sub <- factor(groups[idx])

    ad <- adonis2(
      dist_sub ~ group_sub,
      permutations = perm,
      strata = strata_sub
    )

    results[[i]] <- data.frame(
      Group1 = pair[1],
      Group2 = pair[2],
      F.Model = ad$F[1],
      R2 = ad$R2[1],
      p.value = ad$`Pr(>F)`[1]
    )
  }

  results_df <- bind_rows(results)
  results_df$p.adj <- p.adjust(results_df$p.value, method = p.adjust.method)

  results_df
}

# pairwise season
season_pw <- pairwise_permanova(
  values = df_clean$Phenology,
  groups = df_clean$season,
  strata_factor = df_clean$Transect
```

```
)

print(season_pw)

# pairwise year
year_pw <- pairwise_permanova(
  values = df_clean$Phenology,
  groups = df_clean$Year,
  strata_factor = df_clean$Transect
)

print(year_pw)
```

<u>Phenological Structure: PERMANOVA_AbundanceSpecies</u>

```
library(dplyr)
library(tidyr)
library(vegan)
library(readr)
library(ggplot2)
library(readxl)

df <- read_excel("Clean_Flr_Data_Edit_MEL.xlsx")

# sample id
df$sample_id <- paste(df$Transect, df$season, df$Year, sep = "_")

# community matrix
comm <- group_by(df, sample_id, Transect, season, Year, Species)
```

```
comm <- summarize(comm, present = 1L, .groups = "drop")
comm <- pivot_wider(
  comm,
  names_from = Species,
  values_from = present,
  values_fill = 0
)

comm_mat <- select(comm, -sample_id, -Transect, -season, -Year)
env <- select(comm, sample_id, Transect, season, Year)

# distance
jaccard_dist <- vegdist(comm_mat, method = "jaccard", binary = TRUE)

# permanova
permanova_full <- adonis2(
  jaccard_dist ~ season * Year,
  data = env,
  permutations = 999,
  strata = env$Transect,
  by = "terms"
)

print(permanova_full)
```

Phenological Structure: PERMANOVA_withinSeasonwithSTRATA

```
library(dplyr)
library(tidyr)
library(vegan)
```

```
library(readr)

library(ggplot2)

library(readxl)


df <- read_excel("Clean_Flr_Data_Edit_MEL.xlsx")


# spring

spring_data <- df[df$season == "Spring", ]

spring_groups <- select(spring_data, Transect, Year, Phenology)


dist_spring <- dist(spring_groups$Phenology, method = "euclidean")


permanova_spring <- adonis2(

  dist_spring ~ Transect * Year,

  data = spring_groups,

  permutations = 999,

  strata = spring_groups$Transect

)


print(permanova_spring)


# pairwise function

pairwise_permanova_year <- function(values, groups, strata_factor, perm = 999, p.adjust.method =
"bonferroni") {

  groups <- factor(groups)

  year_pairs <- combn(levels(groups), 2, simplify = TRUE)

  results <- list()


  for (i in 1:ncol(year_pairs)) {
```

```
    pair <- year_pairs[, i]

    idx <- which(groups %in% pair)
    values_sub <- values[idx]
    groups_sub <- factor(groups[idx])
    strata_sub <- strata_factor[idx]

    dist_sub <- dist(values_sub, method = "euclidean")

    adonis_res <- adonis2(
      dist_sub ~ groups_sub,
      permutations = perm,
      strata = strata_sub
    )

    results[[i]] <- data.frame(
      Year1 = pair[1],
      Year2 = pair[2],
      F.Model = adonis_res$F[1],
      R2 = adonis_res$R2[1],
      p.value = adonis_res$`Pr(>F)`[1]
    )
  }

  results_df <- bind_rows(results)
  results_df$p.adj <- p.adjust(results_df$p.value, method = p.adjust.method)

  results_df
}
```

```
# spring pairwise
spring_pw <- pairwise_permanova_year(
  values = spring_groups$Phenology,
  groups = spring_groups$Year,
  strata_factor = spring_groups$Transect
)

print(spring_pw)

# fall
fall_data <- df[df$season == "Fall", ]
fall_groups <- select(fall_data, Transect, Year, Phenology)

dist_fall <- dist(fall_groups$Phenology, method = "euclidean")

permanova_fall <- adonis2(
  dist_fall ~ Transect * Year,
  data = fall_groups,
  permutations = 999,
  strata = fall_groups$Transect
)

print(permanova_fall)

# fall pairwise
fall_pw <- pairwise_permanova_year(
  values = fall_groups$Phenology,
  groups = fall_groups$Year,
```

```
  strata_factor = fall_groups$Transect
)

print(fall_pw)
```

<u>Functional Groups: GrowthForm</u>

```
library(tidyverse)
library(dunn.test)

# load data
df <- read.csv("Clean_Flr_Data_Edit_MEL (1).csv")

growth_col <- names(df)[str_detect(tolower(names(df)), "growth")][1]
cat("Detected growth form column:", growth_col, "\n")

df <- select(df, season, Growth_Form = all_of(growth_col), Phenology)
df <- filter(df, !is.na(season), !is.na(Growth_Form), !is.na(Phenology))

run_growth_test <- function(data, season_name) {
  cat("\n==============================\n")
  cat("Season:", season_name, "\n")
  cat("==============================\n")

  sub <- data[data$season == season_name, ]

  if (n_distinct(sub$Growth_Form) > 1) {
    kw <- kruskal.test(Phenology ~ Growth_Form, data = sub)
    print(kw)
```

```
    if (kw$p.value < 0.05) {
      cat("\nPost-hoc Dunn's Test (Holm correction):\n")
      dunn_res <- dunn.test(sub$Phenology, sub$Growth_Form, method = "holm")
      print(dunn_res)
    } else {
      cat("\nNo significant overall difference among growth forms.\n")
    }
  } else {
    cat("Not enough groups to run the test.\n")
  }
}

run_growth_test(df, "Spring")
run_growth_test(df, "Fall")
```

Functional Groups: SpeciesStatus

```
library(ggplot2)

df <- read.csv("Clean_Flr_Data_Edit_MEL.csv")

## Wilcoxon test (all season)
mw_test <- wilcox.test(Phenology ~ Status, data = df)
mw_test

## Wilcoxon test (Spring)
spring <- df[df$season == "Spring", ]
spring_test <- wilcox.test(Phenology ~ Status, data = spring)
spring_test
```

```
## Wilcoxon test (Fall)
fall <- df[df$season == "Fall", ]
fall_test <- wilcox.test(Phenology ~ Status, data = fall)
fall_test

############### Graph ##########

# spring
ggplot(spring, aes(x = Status, y = Phenology, fill = Status)) +
  geom_boxplot(
    outlier.shape = NA,
    color = "black",
    lwd = 0.8
  ) +
  scale_fill_manual(values = rep("SteelBlue2", length(unique(spring$Status)))) +
  stat_boxplot(geom = "errorbar", width = 0.3) +
  theme_minimal() +
  labs(
    title = "Phenology Index by Species Status (Spring)",
    x = "Species Status",
    y = "Phenology Index"
  ) +
  theme(legend.position = "none")

# fall
ggplot(fall, aes(x = Status, y = Phenology, fill = Status)) +
  geom_boxplot(
    outlier.shape = NA,
    color = "black",
```

```
    lwd = 0.8
  ) +
  scale_fill_manual(values = rep("DarkGoldenrod", length(unique(fall$Status)))) +
  stat_boxplot(geom = "errorbar", width = 0.3) +
  theme_minimal() +
  labs(
    title = "Phenology Index by Species Status (Fall)",
    x = "Species Status",
    y = "Phenology Index"
  ) +
  theme(legend.position = "none")
```

Functional Groups: Duration

```
library(tidyverse)
library(dunn.test)
library(ggplot2)

# load data
df <- read.csv("Clean_Flr_Data_Edit_MEL.csv")

table(df$Duration)

# assumption check
aov_model <- aov(Phenology ~ Duration, data = df)
shapiro.test(residuals(aov_model))
car::leveneTest(Phenology ~ Duration, data = df)

# kruskal test
kruskal.test(Phenology ~ Duration, data = df)
```

```
pairwise.wilcox.test(
  df$Phenology,
  df$Duration,
  p.adjust.method = "BH"
)

# all data plot
ggplot(df, aes(x = Duration, y = Phenology, fill = Duration)) +
  geom_boxplot(outlier.shape = NA, alpha = 0.6) +
  geom_jitter(aes(color = Duration), width = 0.2, size = 2, alpha = 0.8) +
  theme_minimal() +
  labs(
    title = "Phenology Index by Duration (Spring/Fall)",
    x = "Duration Type",
    y = "Phenology Index"
  ) +
  theme(legend.position = "none")

# spring
spring <- df[df$season == "Spring", ]

kruskal.test(Phenology ~ Duration, data = spring)

pairwise.wilcox.test(
  spring$Phenology,
  spring$Duration,
  p.adjust.method = "BH"
)
```

```
ggplot(spring, aes(x = Duration, y = Phenology, fill = Duration)) +
  geom_boxplot(outlier.shape = NA, alpha = 0.6) +
  geom_jitter(aes(color = Duration), width = 0.2, size = 2, alpha = 0.8) +
  theme_minimal() +
  labs(
    title = "Phenology Index by Duration in Spring",
    x = "Duration Type",
    y = "Phenology Index"
  ) +
  theme(legend.position = "none")

# fall
fall <- df[df$season == "Fall", ]

kruskal.test(Phenology ~ Duration, data = fall)

pairwise.wilcox.test(
  fall$Phenology,
  fall$Duration,
  p.adjust.method = "BH"
)

ggplot(fall, aes(x = Duration, y = Phenology, fill = Duration)) +
  geom_boxplot(outlier.shape = NA, alpha = 0.6) +
  geom_jitter(aes(color = Duration), width = 0.2, size = 2, alpha = 0.8) +
  theme_minimal() +
  labs(
    title = "Phenology Index by Duration in Fall",
```

```
    x = "Duration Type",
    y = "Phenology Index"
  ) +
  theme(legend.position = "none")
```

Weather: Fall_MINTemp

```
library(tidyverse)
library(lubridate)
library(car)
library(readr)

# load data
fall <- read_csv(
  "Fall_Data_11-28-2024.csv",
  show_col_types = FALSE
)

fall$DATE <- as.Date(fall$DATE, format = "%m/%d/%Y")
fall$YEAR <- as.integer(fall$YEAR)
fall$TEMP_MN <- as.numeric(fall$TEMP_MN)

fall <- drop_na(fall, DATE, YEAR, TEMP_MN)

# weekly mean
fall_weekly <- mutate(fall, WEEK = isoweek(DATE))
fall_weekly <- group_by(fall_weekly, YEAR, WEEK)
fall_weekly <- summarise(
  fall_weekly,
  mean_temp = mean(TEMP_MN, na.rm = TRUE),
```

```
  .groups = "drop"
)

# weekly line plot
ggplot(fall_weekly, aes(x = WEEK, y = mean_temp, color = factor(YEAR), group = YEAR)) +
  geom_line(linewidth = 1.1) +
  geom_point(size = 1.8, alpha = 0.8) +
  theme_minimal(base_size = 14) +
  labs(
    title = "Fall Weekly Mean Minimum Temperature by Year",
    x = "ISO Week",
    y = "Weekly Mean Minimum Temperature (°F)",
    color = "Year"
  ) +
  theme(legend.position = "bottom")

# anova check
fit_aov <- aov(mean_temp ~ factor(YEAR), data = fall_weekly)
shap_p <- shapiro.test(residuals(fit_aov))$p.value
lev_p <- leveneTest(mean_temp ~ factor(YEAR), data = fall_weekly)[1, "Pr(>F)"]

cat("\nShapiro-Wilk p =", shap_p, "\n")
cat("Levene's p =", lev_p, "\n")

if (shap_p > 0.05 && lev_p > 0.05) {
  cat("\nAssumptions satisfied - you can use ANOVA.\n")
  print(summary(fit_aov))
  print(TukeyHSD(fit_aov))
} else {
```

```r
  cat("\nAssumptions violated - using Kruskal-Wallis test.\n")
  kw_result <- kruskal.test(mean_temp ~ factor(YEAR), data = fall_weekly)
  print(kw_result)

  cat("\nPairwise Wilcoxon tests (Holm-adjusted):\n")
  print(
    pairwise.wilcox.test(
      fall_weekly$mean_temp,
      factor(fall_weekly$YEAR),
      p.adjust.method = "holm",
      exact = FALSE
    )
  )
}

# yearly summary
fall_means2 <- group_by(fall_weekly, YEAR)
fall_means2 <- summarise(
  fall_means2,
  mean_temp = mean(mean_temp, na.rm = TRUE),
  sd_temp = sd(mean_temp, na.rm = TRUE),
  n = n(),
  se_temp = sd_temp / sqrt(n),
  .groups = "drop"
)
fall_means2 <- arrange(fall_means2, YEAR)

print(fall_means2)
```

```
# box plot
ggplot(fall_weekly, aes(x = factor(YEAR), y = mean_temp, fill = factor(YEAR))) +
  geom_boxplot(alpha = 0.7, width = 0.7, color = "gray30", outlier.size = 2) +
  theme_minimal(base_size = 14) +
  labs(
    title = "Fall Weekly Mean Minimum Temperature by Year",
    x = "Year",
    y = "Weekly Mean Minimum Temperature (°F)",
    fill = "Year"
  ) +
  theme(
    legend.position = "none",
    plot.title = element_text(face = "bold", hjust = 0.5)
  )
```

Weather: Fall_DailyAVGTemp

```
library(tidyverse)
library(lubridate)
library(car)
library(readr)

# load data
fall <- read_csv(
  "Fall_Data_11-28-2024.csv",
  show_col_types = FALSE
)

fall$DATE <- as.Date(fall$DATE)
fall$YEAR <- as.integer(fall$YEAR)
```

```r
fall$TEMP_AV <- as.numeric(fall$TEMP_AV)

fall <- drop_na(fall, DATE, YEAR, TEMP_AV)

# weekly mean
fall_weekly <- mutate(fall, WEEK = isoweek(DATE))
fall_weekly <- group_by(fall_weekly, YEAR, WEEK)
fall_weekly <- summarise(
  fall_weekly,
  mean_temp = mean(TEMP_AV, na.rm = TRUE),
  .groups = "drop"
)

# weekly line plot
ggplot(fall_weekly, aes(x = WEEK, y = mean_temp, color = factor(YEAR), group = YEAR)) +
  geom_line(linewidth = 1.1) +
  geom_point(size = 1.8, alpha = 0.8) +
  theme_minimal(base_size = 14) +
  labs(
    title = "Fall Weekly Mean Temperature by Year",
    x = "ISO Week",
    y = "Mean Weekly Temperature (°F)",
    color = "Year"
  ) +
  theme(legend.position = "bottom")

# anova check
fit_aov <- aov(mean_temp ~ factor(YEAR), data = fall_weekly)
shap_p <- shapiro.test(residuals(fit_aov))$p.value
```

```
lev_p <- leveneTest(mean_temp ~ factor(YEAR), data = fall_weekly)[1, "Pr(>F)"]

cat("\nShapiro-Wilk p =", shap_p, "\n")
cat("Levene's p =", lev_p, "\n")

# yearly summary
fall_weekly <- ungroup(fall_weekly)

fall_means <- group_by(fall_weekly, YEAR)
fall_means <- summarise(
  fall_means,
  mean_temp = mean(mean_temp, na.rm = TRUE),
  sd_temp = sd(mean_temp, na.rm = TRUE),
  n = n(),
  se_temp = sd_temp / sqrt(n),
  .groups = "drop"
)
fall_means <- arrange(fall_means, YEAR)

print(fall_means)

# kruskal test
kw_result <- kruskal.test(mean_temp ~ factor(YEAR), data = fall_weekly)
print(kw_result)

pairwise_result <- pairwise.wilcox.test(
  fall_weekly$mean_temp,
  factor(fall_weekly$YEAR),
  p.adjust.method = "holm",
```

```
  exact = FALSE
)

print(pairwise_result)

# box plot
ggplot(fall_weekly, aes(x = factor(YEAR), y = mean_temp, fill = factor(YEAR))) +
  geom_boxplot(alpha = 0.7, width = 0.7, color = "gray30", outlier.size = 2) +
  theme_minimal(base_size = 14) +
  labs(
    title = "Fall Weekly Mean Temperature by Year",
    x = "Year",
    y = "Weekly Mean Temperature (°F)",
    fill = "Year"
  ) +
  theme(
    legend.position = "none",
    plot.title = element_text(face = "bold", hjust = 0.5),
    axis.text.x = element_text(size = 12),
    axis.text.y = element_text(size = 12)
  )
```

Weather: Spring_MinTemp

```
library(tidyverse)
library(lubridate)
library(car)
library(readr)

# load data
```

```
spring <- read_csv(
  "Spring_Data_11-28-2024.csv",
  show_col_types = FALSE
)

spring$DATE <- as.Date(spring$DATE, format = "%m/%d/%Y")
spring$YEAR <- as.integer(spring$YEAR)
spring$TEMP_MN <- as.numeric(spring$TEMP_MN)

spring <- drop_na(spring, DATE, YEAR, TEMP_MN)

# weekly mean
spring_weekly <- mutate(spring, WEEK = isoweek(DATE))
spring_weekly <- group_by(spring_weekly, YEAR, WEEK)
spring_weekly <- summarise(
  spring_weekly,
  mean_temp = mean(TEMP_MN, na.rm = TRUE),
  .groups = "drop"
)

# weekly line plot
ggplot(spring_weekly, aes(x = WEEK, y = mean_temp, color = factor(YEAR), group = YEAR)) +
  geom_line(linewidth = 1.1) +
  geom_point(size = 1.8, alpha = 0.8) +
  theme_minimal(base_size = 14) +
  labs(
    title = "Spring Weekly Mean Minimum Temperature by Year",
    x = "ISO Week",
    y = "Weekly Mean Minimum Temperature (°F)",
```

```
    color = "Year"
  ) +
  theme(legend.position = "bottom")

# anova check
fit_aov <- aov(mean_temp ~ factor(YEAR), data = spring_weekly)
shap_p <- shapiro.test(residuals(fit_aov))$p.value
lev_p <- leveneTest(mean_temp ~ factor(YEAR), data = spring_weekly)[1, "Pr(>F)"]

cat("\nShapiro-Wilk p =", shap_p, "\n")
cat("Levene's p =", lev_p, "\n")

if (shap_p > 0.05 && lev_p > 0.05) {
  cat("\nAssumptions satisfied - use ANOVA.\n")
  print(summary(fit_aov))
  print(TukeyHSD(fit_aov))
} else {
  cat("\nAssumptions violated - using Kruskal-Wallis test.\n")
  kw_result <- kruskal.test(mean_temp ~ factor(YEAR), data = spring_weekly)
  print(kw_result)

  cat("\nPairwise Wilcoxon tests (Holm-adjusted):\n")
  print(
    pairwise.wilcox.test(
      spring_weekly$mean_temp,
      factor(spring_weekly$YEAR),
      p.adjust.method = "holm",
      exact = FALSE
    )
```

```
  )
}

# yearly summary
spring_means2 <- group_by(spring_weekly, YEAR)
spring_means2 <- summarise(
  spring_means2,
  mean_temp = mean(mean_temp, na.rm = TRUE),
  sd_temp = sd(mean_temp, na.rm = TRUE),
  n = n(),
  se_temp = sd_temp / sqrt(n),
  .groups = "drop"
)
spring_means2 <- arrange(spring_means2, YEAR)

print(spring_means2)

# box plot
ggplot(spring_weekly, aes(x = factor(YEAR), y = mean_temp, fill = factor(YEAR))) +
  geom_boxplot(alpha = 0.7, width = 0.7, color = "gray30", outlier.size = 2) +
  theme_minimal(base_size = 14) +
  labs(
    title = "Spring Weekly Mean Minimum Temperature by Year",
    x = "Year",
    y = "Weekly Mean Minimum Temperature (°F)",
    fill = "Year"
  ) +
  theme(
    legend.position = "none",
```

```
    plot.title = element_text(face = "bold", hjust = 0.5),
    axis.text.x = element_text(size = 12),
    axis.text.y = element_text(size = 12)
  )
```

Weather: SpringPrec

```
library(tidyverse)
library(lubridate)
library(car)
library(readxl)

prec_df2 <- read_excel("Spring_Data_11-28-2024_PREC.xlsx")

# clean data
prec_df2$DATE <- as.Date(prec_df2$DATE)
prec_df2$PREC <- as.numeric(prec_df2$PREC)
prec_df2$PREC[is.na(prec_df2$PREC)] <- 0

summary(prec_df2$PREC)

# yearly summary
prec_means_sp <- group_by(prec_df2, YEAR)
prec_means_sp <- summarise(
  prec_means_sp,
  mean_prec = mean(PREC, na.rm = TRUE),
  sd_prec = sd(PREC, na.rm = TRUE),
  n = n(),
  se_prec = sd_prec / sqrt(n)
)
```

```
prec_means_sp <- arrange(prec_means_sp, YEAR)

print(prec_means_sp)

# boxplot
ggplot(prec_df2, aes(x = as.factor(YEAR), y = PREC, fill = as.factor(YEAR))) +
  geom_boxplot(alpha = 0.7) +
  theme_minimal(base_size = 14) +
  labs(
    x = "Year",
    y = "Daily Precipitation (in)",
    title = "Spring Daily Precipitation by Year"
  )

# weekly precipitation
prec_weekly <- mutate(prec_df2, week = isoweek(DATE))
prec_weekly <- group_by(prec_weekly, YEAR, week)
prec_weekly <- summarise(prec_weekly, week_in = sum(PREC, na.rm = TRUE), .groups = "drop")

ggplot(prec_weekly, aes(x = week, y = week_in, color = factor(YEAR), group = YEAR)) +
  geom_line(linewidth = 1.2) +
  geom_point(size = 1.8) +
  theme_minimal(base_size = 14) +
  labs(
    x = "Weeks",
    y = "Weekly precip (in)",
    title = "Spring Weekly Precipitation by Year",
    color = "Year"
  )
```

```
# check structure
prec_df2$DATE <- as.Date(prec_df2$DATE, format = "%m/%d/%Y")
prec_df2$YEAR <- as.integer(prec_df2$YEAR)
prec_df2$PREC <- as.numeric(prec_df2$PREC)

str(prec_df2)
summary(prec_df2)

cat("Data summary:\n")
print(summary(prec_df2$PREC))
cat("\nYears detected:\n")
print(unique(prec_df2$YEAR))
cat("\nNumber of daily records per year:\n")
print(table(prec_df2$YEAR))

# daily plot
prec_df2 <- arrange(prec_df2, DATE)

p <- ggplot(prec_df2, aes(x = DATE, y = PREC, color = factor(YEAR), group = YEAR)) +
  geom_line(linewidth = 1.1) +
  geom_point(size = 1.2, alpha = 0.6) +
  theme_minimal(base_size = 14) +
  labs(
    title = "Spring Daily Precipitation by Year",
    x = "Date",
    y = "Daily Precipitation (in)",
    color = "Year"
  ) +
```

```
  theme(legend.position = "bottom")

ggsave("spring_daily_temp_by_year.png", p, width = 8, height = 5, dpi = 300)

# anova assumptions
fit_aov_prec_sp <- aov(PREC ~ factor(YEAR), data = prec_df2)

shap <- shapiro.test(residuals(fit_aov_prec_sp))
lev <- leveneTest(PREC ~ factor(YEAR), data = prec_df2)

shap_p <- shap$p.value
lev_p <- lev[1, "Pr(>F)"]

cat("\nChecking ANOVA assumptions...\n")
cat("\nShapiro-Wilk p =", shap_p, "\n")
cat("Levene's p =", lev_p, "\n")

# kruskal test
prec_weekly <- mutate(prec_df2, week = isoweek(DATE))
prec_weekly <- group_by(prec_weekly, YEAR, week)
prec_weekly <- summarise(
  prec_weekly,
  weekly_prec = sum(PREC, na.rm = TRUE),
  .groups = "drop"
)

kruskal.test(weekly_prec ~ factor(YEAR), data = prec_weekly)

pairwise.wilcox.test(
```

```
  prec_weekly$weekly_prec,
  factor(prec_weekly$YEAR),
  p.adjust.method = "holm",
  exact = FALSE
)
```

Weather: Spring_AVGTemp

```
library(tidyverse)
library(lubridate)
library(car)
library(readr)

# load data
spring <- read_csv(
  "Spring_Data_11-28-2024.csv",
  show_col_types = FALSE
)

spring$DATE <- as.Date(spring$DATE)
spring$YEAR <- as.integer(spring$YEAR)
spring$TEMP_AV <- as.numeric(spring$TEMP_AV)

spring <- drop_na(spring, DATE, YEAR, TEMP_AV)

# weekly mean
spring_weekly <- mutate(spring, WEEK = isoweek(DATE))
spring_weekly <- group_by(spring_weekly, YEAR, WEEK)
spring_weekly <- summarise(
  spring_weekly,
  mean_temp = mean(TEMP_AV, na.rm = TRUE),
```

```
  .groups = "drop"
)

# weekly line plot
ggplot(spring_weekly, aes(x = WEEK, y = mean_temp, color = factor(YEAR), group = YEAR)) +
  geom_line(linewidth = 1.1) +
  geom_point(size = 1.8, alpha = 0.8) +
  theme_minimal(base_size = 14) +
  labs(
    title = "Spring Weekly Mean Temperature by Year",
    x = "ISO Week",
    y = "Mean Weekly Temperature (°F)",
    color = "Year"
  ) +
  theme(legend.position = "bottom")

# anova check
fit_aov <- aov(mean_temp ~ factor(YEAR), data = spring_weekly)
shap_p <- shapiro.test(residuals(fit_aov))$p.value
lev_p <- leveneTest(mean_temp ~ factor(YEAR), data = spring_weekly)[1, "Pr(>F)"]

cat("\nShapiro-Wilk p =", shap_p, "\n")
cat("Levene's p =", lev_p, "\n")

# yearly summary
spring_weekly <- ungroup(spring_weekly)

spring_means <- group_by(spring_weekly, YEAR)
spring_means <- summarise(
```

```r
  spring_means,
  mean_temp = mean(mean_temp, na.rm = TRUE),
  sd_temp = sd(mean_temp, na.rm = TRUE),
  n = n(),
  se_temp = sd_temp / sqrt(n)
)
spring_means <- arrange(spring_means, YEAR)

print(spring_means)

# kruskal test
kw_result <- kruskal.test(mean_temp ~ factor(YEAR), data = spring_weekly)
print(kw_result)

pairwise_result <- pairwise.wilcox.test(
  spring_weekly$mean_temp,
  factor(spring_weekly$YEAR),
  p.adjust.method = "holm",
  exact = FALSE
)

print(pairwise_result)

# box plot
ggplot(spring_weekly, aes(x = factor(YEAR), y = mean_temp, fill = factor(YEAR))) +
  geom_boxplot(alpha = 0.7, width = 0.7, color = "gray30", outlier.size = 2) +
  theme_minimal(base_size = 14) +
  labs(
    title = "Spring Weekly Mean Temperature by Year",
```

```
    x = "Year",
    y = "Weekly Mean Temperature (°F)",
    fill = "Year"
  ) +
  theme(
    legend.position = "none",
    plot.title = element_text(face = "bold", hjust = 0.5),
    axis.text.x = element_text(size = 12),
    axis.text.y = element_text(size = 12)
  )
```

Weather: Snow

```
library(tidyverse)
library(lubridate)
library(car)
library(readr)

# load data
winter <- read_csv(
  "Winter_Data_11-28-2024.csv",
  show_col_types = FALSE
)

winter$DATE <- as.Date(winter$DATE, format = "%m/%d/%Y")
winter$YEAR <- as.integer(winter$YEAR)
winter$SNOW_WC <- as.numeric(winter$SNOW_WC)

winter <- drop_na(winter, DATE, YEAR, SNOW_WC)

# weekly mean
```

```
winter_weekly <- mutate(winter, WEEK = isoweek(DATE))
winter_weekly <- group_by(winter_weekly, YEAR, WEEK)
winter_weekly <- summarise(
  winter_weekly,
  mean_snow = mean(SNOW_WC, na.rm = TRUE),
  .groups = "drop"
)

# weekly line plot
ggplot(winter_weekly, aes(x = WEEK, y = mean_snow, color = factor(YEAR), group = YEAR)) +
  geom_line(linewidth = 1.1) +
  geom_point(size = 1.8, alpha = 0.8) +
  theme_minimal(base_size = 14) +
  labs(
    title = "Winter Weekly Mean Snow Water Content by Year",
    x = "ISO Week",
    y = "Weekly Mean SNOW_WC (inches)",
    color = "Year"
  ) +
  theme(legend.position = "bottom")

# anova check
fit_aov <- aov(mean_snow ~ factor(YEAR), data = winter_weekly)
shap_p <- shapiro.test(residuals(fit_aov))$p.value
lev_p <- leveneTest(mean_snow ~ factor(YEAR), data = winter_weekly)[1, "Pr(>F)"]

cat("\nShapiro-Wilk p =", shap_p, "\n")
cat("Levene's p =", lev_p, "\n")
```

```
# choose test
if (shap_p > 0.05 && lev_p > 0.05) {
  cat("\nAssumptions satisfied - using one-way ANOVA.\n\n")
  print(summary(fit_aov))
  print(TukeyHSD(fit_aov))
} else {
  cat("\nAssumptions violated - using Kruskal-Wallis test.\n\n")
  kw_result <- kruskal.test(mean_snow ~ factor(YEAR), data = winter_weekly)
  print(kw_result)

  cat("\nPairwise Wilcoxon tests (Holm-adjusted):\n")
  print(
    pairwise.wilcox.test(
      winter_weekly$mean_snow,
      factor(winter_weekly$YEAR),
      p.adjust.method = "holm",
      exact = FALSE
    )
  )
}

# yearly summary
snow_means <- group_by(winter_weekly, YEAR)
snow_means <- summarise(
  snow_means,
  mean_snow = mean(mean_snow, na.rm = TRUE),
  sd_snow = sd(mean_snow, na.rm = TRUE),
  n = n(),
  se_snow = sd_snow / sqrt(n),
```

```
  .groups = "drop"
)
snow_means <- arrange(snow_means, YEAR)

print(snow_means)

# weekly box plot
ggplot(winter_weekly, aes(x = factor(YEAR), y = mean_snow, fill = factor(YEAR))) +
  geom_boxplot(alpha = 0.7, width = 0.7, color = "gray30", outlier.size = 2) +
  theme_minimal(base_size = 14) +
  labs(
    title = "Winter Weekly Mean Snow Water Content by Year",
    x = "Year",
    y = "Weekly Mean SNOW_WC (inches)",
    fill = "Year"
  ) +
  theme(
    legend.position = "none",
    plot.title = element_text(face = "bold", hjust = 0.5)
  )

# daily box plot
ggplot(winter, aes(x = factor(YEAR), y = SNOW_WC, fill = factor(YEAR))) +
  geom_boxplot(alpha = 0.7, width = 0.7, color = "gray30", outlier.size = 2) +
  theme_minimal(base_size = 14) +
  labs(
    title = "Winter Daily Snow Water Content by Year",
    x = "Year",
    y = "Daily SNOW_WC (inches)",
```

```
    fill = "Year"
  ) +
  theme(
    legend.position = "none",
    plot.title = element_text(face = "bold", hjust = 0.5)
  )
```

Weather: Precipitation

```
library(tidyverse)
library(lubridate)
library(car)
library(readxl)
library(readr)

# load data
prec_df <- read_excel("Fall_Data_11-28-2024_PREC.xlsx")

prec_df$DATE <- as.Date(prec_df$DATE)
prec_df$PREC <- as.numeric(prec_df$PREC)
prec_df$PREC[is.na(prec_df$PREC)] <- 0

summary(prec_df$PREC)

# yearly summary
prec_means <- group_by(prec_df, YEAR)
prec_means <- summarise(
  prec_means,
  mean_prec = mean(PREC, na.rm = TRUE),
  sd_prec = sd(PREC, na.rm = TRUE),
  n = n(),
```

```
  se_prec = sd_prec / sqrt(n)
)
prec_means <- arrange(prec_means, YEAR)

print(prec_means)

# daily box plot
ggplot(prec_df, aes(x = as.factor(YEAR), y = PREC, fill = as.factor(YEAR))) +
  geom_boxplot(alpha = 0.7) +
  theme_minimal(base_size = 14) +
  labs(
    x = "Year",
    y = "Daily Precipitation (in)",
    title = "Fall Daily Precipitation by Year"
  )

# weekly precipitation
prec_weekly_fall <- mutate(prec_df, week = isoweek(DATE))
prec_weekly_fall <- group_by(prec_weekly_fall, YEAR, week)
prec_weekly_fall <- summarise(
  prec_weekly_fall,
  week_in = sum(PREC, na.rm = TRUE),
  .groups = "drop"
)

ggplot(prec_weekly_fall, aes(x = week, y = week_in, color = factor(YEAR), group = YEAR)) +
  geom_line(linewidth = 1.2) +
  geom_point(size = 1.8) +
  theme_minimal(base_size = 14) +
```

```
  labs(
    x = "Weeks",
    y = "Weekly precip (in)",
    title = "Fall Weekly Precipitation by Year",
    color = "Year"
  )

# weekly totals
prec_weekly_fall <- mutate(prec_df, week = isoweek(DATE))
prec_weekly_fall <- group_by(prec_weekly_fall, YEAR, week)
prec_weekly_fall <- summarise(
  prec_weekly_fall,
  weekly_prec = sum(PREC, na.rm = TRUE),
  .groups = "drop"
)

prec_weekly_fall$YEAR <- as.integer(prec_weekly_fall$YEAR)
prec_weekly_fall$weekly_prec <- as.numeric(prec_weekly_fall$weekly_prec)

cat("Data summary:\n")
print(summary(prec_weekly_fall$weekly_prec))
cat("\nYears detected:\n")
print(unique(prec_weekly_fall$YEAR))
cat("\nNumber of daily records per year:\n")
print(table(prec_weekly_fall$YEAR))

# anova check
cat("\nChecking ANOVA assumptions...\n")
```

```
fit_aov_prec <- aov(weekly_prec ~ factor(YEAR), data = prec_weekly_fall)
shap <- shapiro.test(residuals(fit_aov_prec))
lev <- leveneTest(PREC ~ factor(YEAR), data = prec_df)

shap_p <- shap$p.value
lev_p <- lev[1, "Pr(>F)"]

cat("\nShapiro-Wilk p =", shap_p, "\n")
cat("Levene's p =", lev_p, "\n")

# weekly mean summary by year
prec_weekly_mean <- mutate(prec_df, week = isoweek(DATE))
prec_weekly_mean <- group_by(prec_weekly_mean, YEAR, week)
prec_weekly_mean <- summarise(
  prec_weekly_mean,
  weekly_total = sum(PREC, na.rm = TRUE),
  .groups = "drop"
)

prec_weekly_mean <- group_by(prec_weekly_mean, YEAR)
prec_weekly_mean <- summarise(
  prec_weekly_mean,
  mean_weekly_prec = mean(weekly_total, na.rm = TRUE),
  sd_weekly_prec = sd(weekly_total, na.rm = TRUE),
  n_weeks = n(),
  se_weekly_prec = sd_weekly_prec / sqrt(n_weeks),
  .groups = "drop"
)
prec_weekly_mean <- arrange(prec_weekly_mean, YEAR)
```

```
print(prec_weekly_mean)


# kruskal test
kruskal.test(weekly_prec ~ factor(YEAR), data = prec_weekly_fall)


pairwise.wilcox.test(
  prec_weekly_fall$weekly_prec,
  factor(prec_weekly_fall$YEAR),
  p.adjust.method = "holm",
  exact = FALSE
)
```

Linear Mixed-Models

```
library(lme4)
library(lmerTest)
library(readr)


# read data
dat <- read_csv("Downloads/spring_species_LMM_dataset_all_species.csv")


# prep
dat$Year <- factor(dat$Year)
dat$Species <- factor(dat$Species)



# null models


mod_null_species <- lmer(
  pheno_mean ~ 1 + (1 | Species),
```

```
  data = dat,
  REML = FALSE
)

mod_null_species_year <- lmer(
  pheno_mean ~ 1 + (1 | Species) + (1 | Year),
  data = dat,
  REML = FALSE
)

summary(mod_null_species)
summary(mod_null_species_year)


# climate models (Species)

mod_precip_total_species <- lmer(
  pheno_mean ~ precip_total + (1 | Species),
  data = dat,
  REML = FALSE
)

mod_forcing_DD_above0_species <- lmer(
  pheno_mean ~ forcing_DD_above0 + (1 | Species),
  data = dat,
  REML = FALSE
)

mod_chilling_DD_below0_species <- lmer(
```

```
  pheno_mean ~ chilling_DD_below0 + (1 | Species),
  data = dat,
  REML = FALSE
)

mod_freezing_days_species <- lmer(
  pheno_mean ~ freezing_days + (1 | Species),
  data = dat,
  REML = FALSE
)

mod_mean_TAVG_species <- lmer(
  pheno_mean ~ mean_TAVG + (1 | Species),
  data = dat,
  REML = FALSE
)



# climate models (Species and Year)

mod_precip_total_species_year <- lmer(
  pheno_mean ~ precip_total + (1 | Species) + (1 | Year),
  data = dat,
  REML = FALSE
)

mod_forcing_DD_above0_species_year <- lmer(
  pheno_mean ~ forcing_DD_above0 + (1 | Species) + (1 | Year),
  data = dat,
```

```
  REML = FALSE
)

mod_chilling_DD_below0_species_year <- lmer(
  pheno_mean ~ chilling_DD_below0 + (1 | Species) + (1 | Year),
  data = dat,
  REML = FALSE
)

mod_freezing_days_species_year <- lmer(
  pheno_mean ~ freezing_days + (1 | Species) + (1 | Year),
  data = dat,
  REML = FALSE
)

mod_mean_TAVG_species_year <- lmer(
  pheno_mean ~ mean_TAVG + (1 | Species) + (1 | Year),
  data = dat,
  REML = FALSE
)


# AIC check

AIC(
  mod_null_species,
  mod_precip_total_species,
  mod_forcing_DD_above0_species,
  mod_chilling_DD_below0_species,
```

```
  mod_freezing_days_species,
  mod_mean_TAVG_species
)

AIC(
  mod_null_species_year,
  mod_precip_total_species_year,
  mod_forcing_DD_above0_species_year,
  mod_chilling_DD_below0_species_year,
  mod_freezing_days_species_year,
  mod_mean_TAVG_species_year
)


# function for results

get_row <- function(mod, model_name, structure_name) {
  s <- summary(mod)$coefficients
  data.frame(
    model = model_name,
    structure = structure_name,
    predictor = rownames(s)[2],
    estimate = s[2, 1],
    se = s[2, 2],
    t = s[2, 3],
    p = s[2, 5],
    AIC = AIC(mod)
  )
}
```

```
# results

res <- rbind(
  get_row(mod_precip_total_species, "precip_total", "Species"),
  get_row(mod_forcing_DD_above0_species, "forcing_DD_above0", "Species"),
  get_row(mod_chilling_DD_below0_species, "chilling_DD_below0", "Species"),
  get_row(mod_freezing_days_species, "freezing_days", "Species"),
  get_row(mod_mean_TAVG_species, "mean_TAVG", "Species"),
  get_row(mod_precip_total_species_year, "precip_total", "Species+Year"),
  get_row(mod_forcing_DD_above0_species_year, "forcing_DD_above0", "Species+Year"),
  get_row(mod_chilling_DD_below0_species_year, "chilling_DD_below0", "Species+Year"),
  get_row(mod_freezing_days_species_year, "freezing_days", "Species+Year"),
  get_row(mod_mean_TAVG_species_year, "mean_TAVG", "Species+Year")
)

res <- res[order(res$structure, res$AIC), ]
print(res)

write_csv(res, "spring_species_LMM_results_all_models.csv")

# AIC tables

aic_species <- as.data.frame(AIC(
  mod_null_species,
  mod_precip_total_species,
  mod_forcing_DD_above0_species,
```

```
  mod_chilling_DD_below0_species,
  mod_freezing_days_species,
  mod_mean_TAVG_species
))
aic_species$model <- rownames(aic_species)
aic_species$structure <- "Species"
aic_species$deltaAIC <- aic_species$AIC - min(aic_species$AIC)
aic_species <- aic_species[order(aic_species$AIC), ]

aic_species_year <- as.data.frame(AIC(
  mod_null_species_year,
  mod_precip_total_species_year,
  mod_forcing_DD_above0_species_year,
  mod_chilling_DD_below0_species_year,
  mod_freezing_days_species_year,
  mod_mean_TAVG_species_year
))
aic_species_year$model <- rownames(aic_species_year)
aic_species_year$structure <- "Species+Year"
aic_species_year$deltaAIC <- aic_species_year$AIC - min(aic_species_year$AIC)
aic_species_year <- aic_species_year[order(aic_species_year$AIC), ]

aic_tab <- rbind(aic_species, aic_species_year)
aic_tab <- aic_tab[order(aic_tab$structure, aic_tab$AIC), ]

print(aic_tab)

write_csv(aic_tab, "spring_species_LMM_AIC_tables.csv")
```
